# DNA end-resection is stimulated by an interaction between BRCA1 exon 11 and TOPBP1

**DOI:** 10.1038/s44319-026-00904-3

**Published:** 2026-08-27

**Authors:** Marta San Martín Alonso, Rosalie A Kampen, Anne Schreuder, Daniel Salas, Arnoud H de Ru, Veronica Garzero, Romy L S Mesman, Román González-Prieto, Peter A van Veelen, Sylvie M Noordermeer

**Affiliations:** 1 Department of Human Genetics, Leiden University Medical Center, Leiden, The Netherlands; 2 Oncode Institute, Utrecht, The Netherlands; 3 Department of Cell and Chemical Biology, Leiden University Medical Center, Leiden, The Netherlands; 4 Center for Proteomics and Metabolomics, Leiden University Medical Center, Leiden, The Netherlands; 5 Present Address: Experimental Therapeutics Programme, Spanish National Cancer Research Centre (CNIO), Madrid, Spain; 6 Present Address: HmARbcn Cancer Immunotherapy Clinical Research Unit, CNIO, Madrid, Spain; 7 Present Address: Centro Andaluz de Biología Molecular & Medicina Regenerativa (CABIMER), Universidad de Sevilla-CSIC-Universidad Pablo de Olavide-Junta de Andalucía, Sevilla, Spain

**Keywords:** DNA Replication, Recombination & Repair

## Abstract

Approximately 60% of the tumour suppressor protein BRCA1 is encoded by a single exon. Many tumours carry mutations in this exon, often resulting in exon skipping and thus a protein of a severely reduced size. Although none of the well-described protein domains of BRCA1 are encoded by this exon 11, the isoform lacking this part shows a hypomorphic activity in homologous recombination. To better understand the function of this large exon, we performed proteomic analyses to identify interaction partners via this part of the protein. Here, we report a DNA damage- and phospho-dependent interaction of TOPBP1 with the protein region of BRCA1 encoded by exon 11. Mechanistically, this interaction is required for BRCA1’s role in end-resection during homologous recombination. In contrast, the interaction is not required for TOPBP1’s role in ATR activation. Our data provide novel mechanistic insight into the function of this poorly characterized part of the BRCA1 protein.

## Synopsis

The region of BRCA1 encoded by exon 11 binds in a damage-dependent manner to TOPBP1. This interaction stimulates BRCA1’s role in DNA end-resection but is not required for TOPBP1’s role in ATR activation.

- BRCA1 forms a complex with TOPBP1 via two independent motifs;
- The BRCA1 region encoded by exon 11 binds TOPBP1;
- The interaction of BRCA1 exon 11 with TOPBP1 stimulates DNA end-resection;

## Abstract (web summary)

The region of BRCA1 encoded by exon 11 binds in a damage-dependent manner to TOPBP1. This interaction stimulates BRCA1’s role in DNA end-resection but is not required for TOPBP1’s role in ATR activation.

## Graphical abstract

**Figure:**
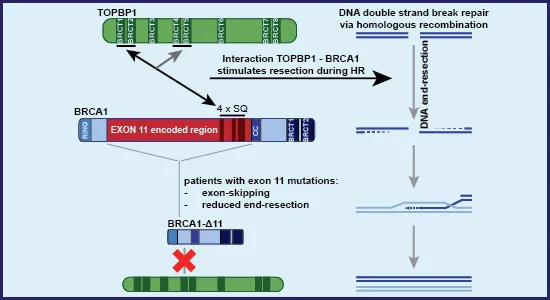

## Introduction

Cells are constantly exposed to different exogenous and endogenous agents that can damage the DNA molecule. One of the most toxic lesions in the DNA is the DNA double-stranded break (DSB). Failure to repair such breaks can lead to genomic instability, an important cause of cancer. Fortunately, cells have evolved different ways to detect and repair damaged DNA to maintain genetic integrity (Tubbs and Nussenzweig, 2017). The cell is equipped with multiple repair pathways to overcome DSBs, varying in their efficiency and accuracy: classical non-homologous end joining (cNHEJ), alternative end joining (aEJ), single strand annealing (SSA), and homologous recombination (HR) (Scully et al, 2019).

HR utilizes the intact sister chromatid to copy the genetic information during repair, resulting in high fidelity, error-free repair. This pathway is activated by DNA end-resection of the broken ends to expose ssDNA stretches that can be used for homology search and templated repair. BRCA1 is an important player in HR stimulating both resection and the homology search (Tarsounas and Sung, 2020). Hereditary BRCA1 mutations predispose to breast and ovarian cancers (Hall, 1990; Lord and Ashworth, 2016; Couch et al, 2015), but mutations also frequently arise in sporadic tumours (Kandoth et al, 2013). Approximately 30% of the mutations in BRCA1 target exon 10—historically known as exon 11 and hence also referred to as exon 11 in this manuscript—a large exon encoding for about 60% of the protein (Rebbeck et al, 2015; Risch et al, 2006). Interestingly, these mutations frequently result in exon skipping (resulting in *BRCA1-Δ11*) or usage of an alternative splice donor site (*BRCA1-11q*). Both transcripts are naturally occurring isoforms coding for proteins lacking a large part of the wild-type protein although retaining the well-studied functional domains of BRCA1 (including the RING, coiled-coil and BRCT domains), suggesting residual functionality (Colombo et al, 2014; Orban and Olah, 2001). Indeed, compared to BRCA1-deficiency, which is embryonically lethal, mice carrying BRCA1-Δ11 alleles are viable in a *TP53*^−/−^ background, although suffering from increased tumorigenesis (Bachelier et al, 2003; Kim et al, 2006; Xu et al, 2001; Yueh et al, 2024). Moreover, previous studies have demonstrated that mutations in exon 11 result in hypomorphic phenotypes such as partially reduced HR efficiency (reduced RAD51 and RPA recruitment to DNA damage sites) and resistance to therapy (Nacson et al, 2020; Wang et al, 2016b). Better understanding of the functional role of exon 11 in the maintenance of genome stability is therefore essential to improve prediction of disease progression and therapy response of patients with tumours carrying exon 11 mutations.

In this study, we identified interaction partners of the region in BRCA1 encoded by exon 11. Among these interactors was TOPBP1, which showed a damage-dependent interaction with this region. TOPBP1 is a protein consisting of eight BRCT domains and was originally identified as a topoisomerase 2 β interactor, hence its name (Yamane et al, 1997). TOPBP1 is recruited to DSBs and single stranded DNA (ssDNA) directly, where it can activate the ataxia telangiectasia and Rad3-related (ATR) enzyme. ATR is involved in the activation of DNA damage checkpoints, initiating cell cycle arrest to slow down the replication fork and repair the damaged DNA (Kumagai et al, 2006; Mordes et al, 2008; Saldivar et al, 2017). TOPBP1 is also capable to interact with NBS1, driving efficient HR (Morishima et al, 2007) and is involved in balancing 53BP1 and BRCA1 interplay to stimulate HR (Liu et al, 2017). Moreover, previous studies have identified an interaction between BRIP1 (also known as FANCJ or BACH1) and TOPBP1, specifically through the C-terminal BRCT domains (BRCT 7 and 8) of TOPBP1 (Gong et al, 2010). BRIP1 also interacts with the BRCT domains of BRCA1 (Cantor et al, 2001), and hence it has been shown that BRCA1, BRIP1 and TOPBP1 are present in the same protein complex (Greenberg et al, 2006). Interestingly, our data indicate that the association between BRCA1 and TOPBP1 is only marginally affected when BRCA1 harbours a mutation that impairs its interaction with BRIP1, indicating that TOPBP1 and BRCA1 also bind in a BRIP1-independent setting. Here, we show that exon 11 of BRCA1 provides a second binding interface to TOPBP1, and that this interaction stimulates the role of BRCA1 in DNA end-resection.

## Results and discussion

### BRCA1 binds TOPBP1 through a region encoded by exon 11

**Figure 1:**
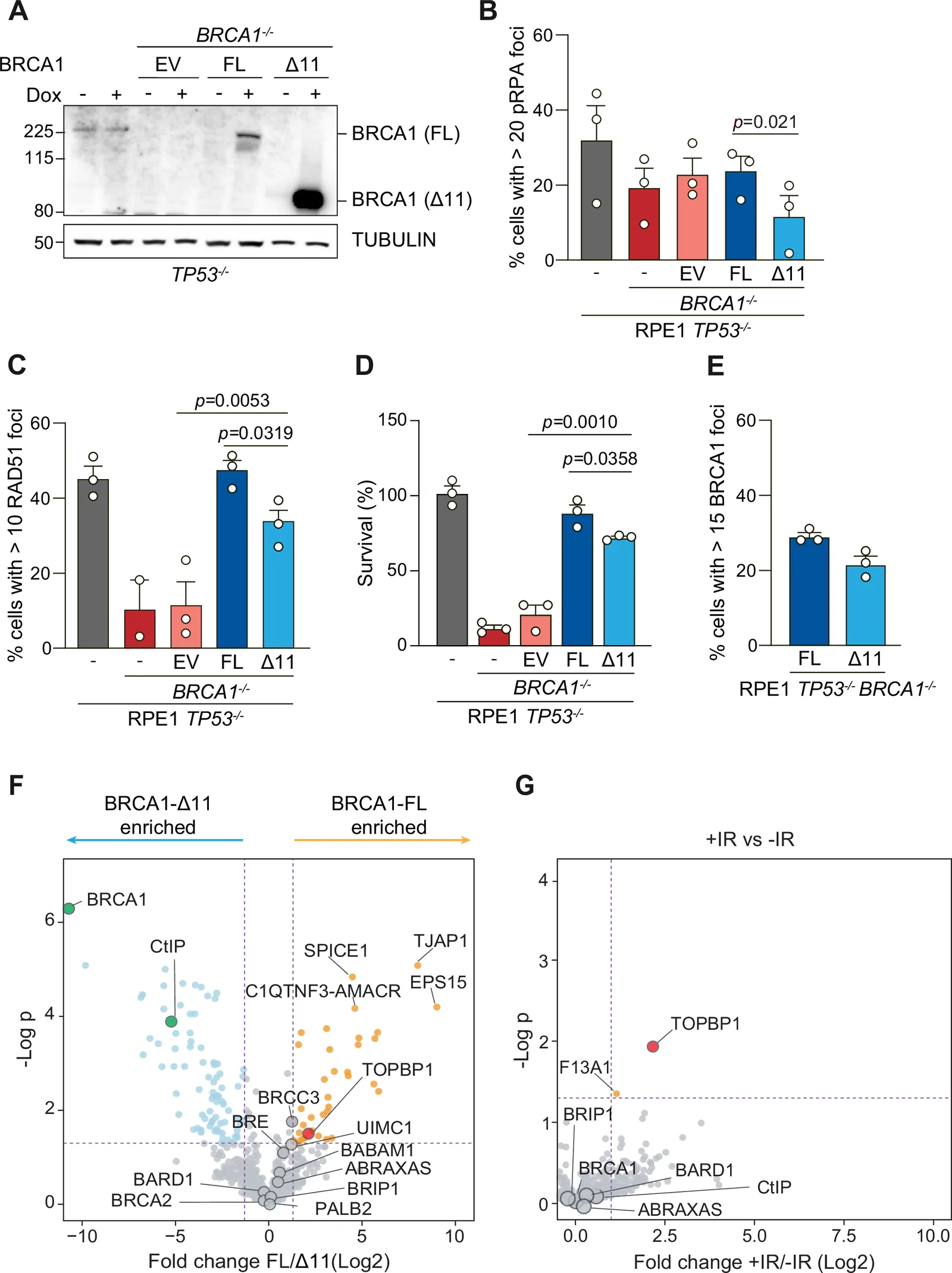
BRCA1 binds TOPBP1 through its exon 11 encoded region. (**A**) Lysates of RPE1 hTERT cell lines either *TP53*^−/−^ or *TP53*^−/−^ *BRCA1*^−/−^ virally reconstituted with a dox-inducible BRCA1-TY-1 cDNA (FL), BRCA1-TY-1 exon11-less (∆11) cDNA or an empty vector (EV) control analyzed by western blotting to indicate BRCA1 variant expression 24 h post dox induction (1 μg/mL). (**B**) *TP53*^−/−^ or *TP53*^−/−^ *BRCA1*^−/−^ virally reconstituted with dox-inducible FL BRCA1-TY-1 or BRCA1-TY-1-∆11 were irradiated with 10 Gy and 4 h post-IR analyzed by IF microscopy to detect p-RPA (RPA32 pS4/8) foci formation (*n* = 3, mean + SEM (biological replicates), paired *t* test, *P* values are shown). A minimum of 100 cells were counted per replicate. (**C**) Same cells used in (**A**, **B**) were irradiated as in (**B**) and IF microscopy was performed to quantify RAD51 foci (*n* = 2 (untransduced BRCA1^−/−^ cells) or 3 (other samples), mean + SEM (biological replicates), paired *t* test, *P* values are shown). A minimum of 100 cells were counted per replicate. (**D**) Clonogenic survival assay with continuous treatment of 16 nM olaparib and 1 μg/mL dox of the same cell lines described in (**A**). Data is normalized to cells treated with dox, but not treated with PARPi (*n* = 3, mean + SEM (biological replicates), paired *t* test, *P* values are shown). (**E**) Same cells used in (**A**, **B**) were irradiated as in (**B**) and IF microscopy was performed to quantify BRCA1 foci (*n* = 3, mean + SEM (biological replicates)). A minimum of 100 cells were counted per replicate. (**F**) Volcano plot depicting statistical differences in binding proteins identified by MS proteomic analysis upon TY-1 IP of RPE1 hTERT *TP53*^−/−^ *BRCA1*^−/−^ cells virally reconstituted with dox-inducible BRCA1-FL or BRCA1-∆11 1 h post 5 Gy IR (*n* = 3 (biological replicates), fold-changes were determined by Perseus software, two-tiered *t* tests, *P* value < 0.05 was considered significant). (**G**) Volcano plot depicting statistical differences in binding proteins identified by MS proteomic analysis upon TY-1 IP of mock- or IR-treated (1 h post 5 Gy) RPE1 hTERT *TP53*^−/−^ cells with an endogenous BRCA1-TY-1 tag (*n* = 3 (biological replicates), fold-changes were determined by Perseus software, two-tiered *t* tests, *P* value < 0.05 was considered significant). Source data are available online for this figure.

**Figure EV1:**
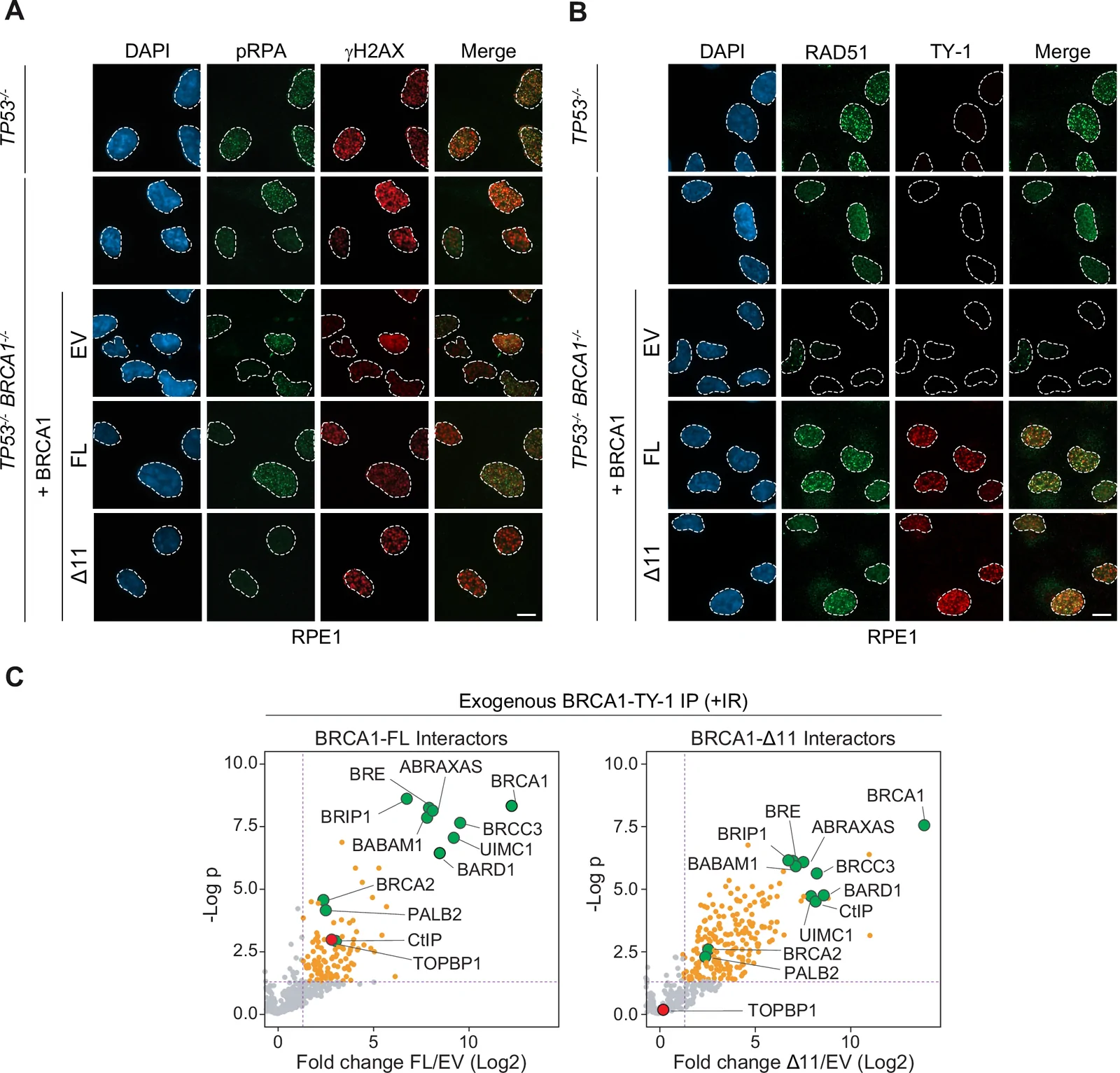
BRCA1 exon 11 mediates HR and interacts directly to TOPBP1 (related to Fig. 1). Representative microscopy images of p-RPA (**A**) or RAD51 foci (**B**) quantified in Fig. 1B,C, respectively (scale bar 10 µm). (**C**) Volcano plots depicting statistical differences in binding proteins identified by MS proteomic analysis upon TY-1 IP of RPE1 hTERT *TP53*^−/−^ *BRCA1*^−/−^ cells virally reconstituted with dox-inducible BRCA1-FL or BRCA1-∆11 1 h post 5 Gy IR compared to EV 1 h post 5 Gy IR (*n* = 3 (biological replicates), fold-changes were determined by Perseus software, two-tiered *t* tests, *P* value < 0.05 was considered significant). Source data are available online for this figure

To better understand the function of exon 11 of BRCA1 during DSB repair, we aimed to identify proteins that bind BRCA1 specifically through the protein region encoded by this exon 11. For this, we generated an isogenic cell line model expressing either full length (FL) BRCA1 or a variant lacking exon 11 (Δ11) by virally reconstituting RPE1 hTERT *TP53*^−/−^ *BRCA1*^−/−^ cells with doxycycline (dox) inducible versions of the two BRCA1 protein variants with C-terminal triple TY-1 tags (or an empty vector control) (Fig. 1A). Compared to FL BRCA1, BRCA1-Δ11 was expressed at higher levels, as has been observed before in other cell line models and clinical samples (Nacson et al, 2018; Wang et al, 2016b). Phenotypic characterization of these cell lines confirmed the hypomorphic effect of BRCA1-Δ11 (Nacson et al, 2020; Wang et al, 2016b), showing reduced DNA end-resection (Figs. 1B and  EV1A), reduced RAD51 foci formation (Figs. 1C and  EV1B), and mild PARPi sensitivity (Fig. 1D). Moreover, BRCA1- Δ11 was efficiently recruited to sites of DNA damage, comparable to FL BRCA1, confirming what has been shown before and indicating that the nuclear localisation signals (NSL) within exon 11 are not required for its nuclear localisation (Fig. 1E) (Fabbro et al, 2002; Qin et al, 2011).

To identify interaction partners of exon 11’s coding region, we performed immunoprecipitation (IP) experiments followed by liquid chromatography-tandem mass spectrometry (LC-MS/MS) (IP-MS) analyses. After irradiating the cells to induce DSBs, we purified FL BRCA1 or BRCA1-Δ11 and their interactors using antibodies against the TY-1 tag. Using this approach, we identified many well-described interactors that bind BRCA1 via domains outside of exon 11 (e.g., BARD1, PALB2, ABRAXAS, BRCA2, CtIP, BRIP1). Indeed, all these interactors were retained in BRCA1-∆11 (Figs. 1F and EV1C; Dataset EV1). Importantly, we observed a set of proteins in our FL BRCA1 IP which were not identified in the BRCA1-Δ11 IP. Given the low expression level of FL BRCA1 compared to BRCA1-Δ11, these are strong candidates to be exon 11 specific interactors.

**Figure EV2:**
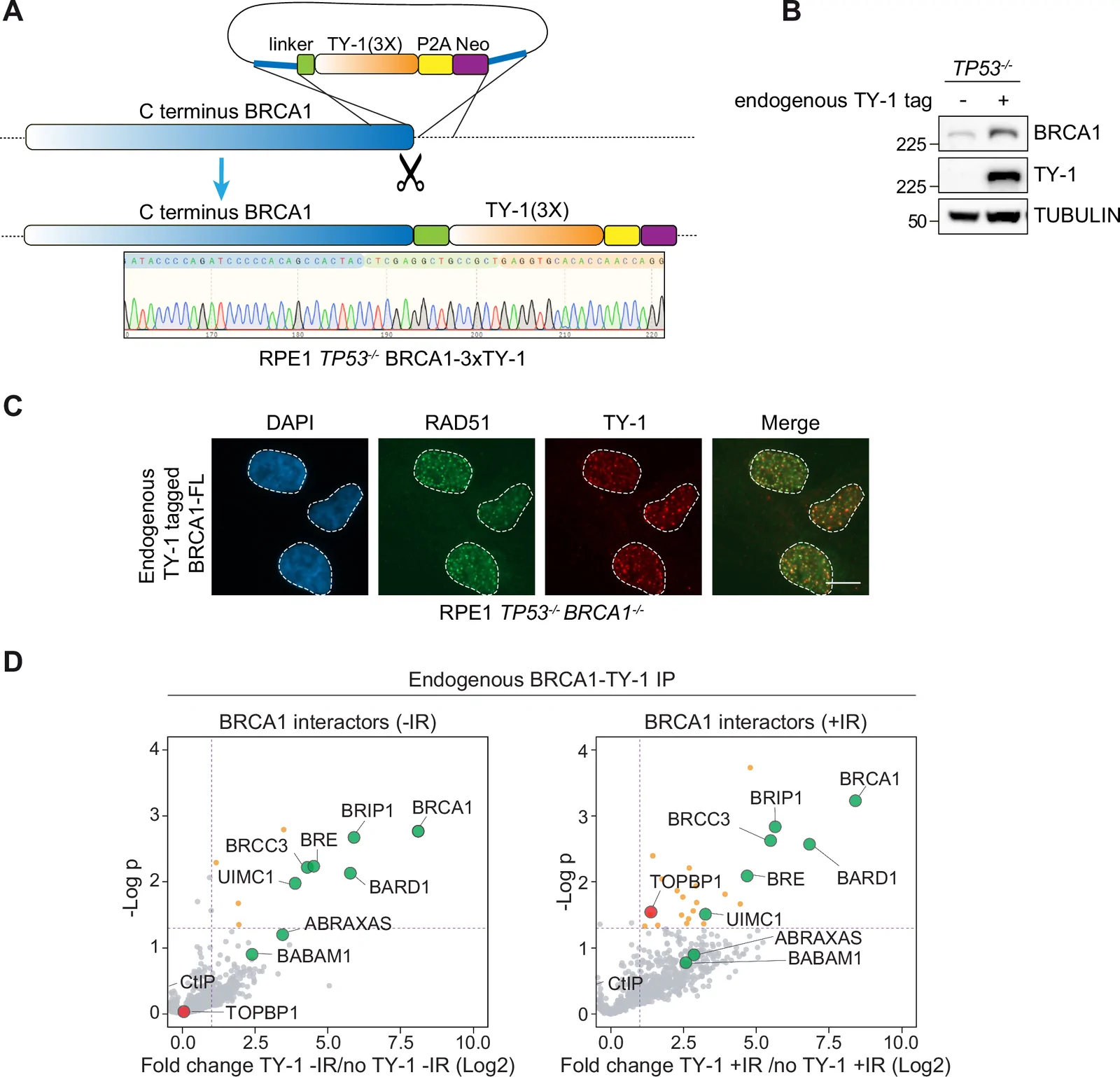
Generation of an endogenous BRCA1-TY-1 cell line and analyses of its interactors by mass spectrometry (related to Fig. 1). (**A**) Schematic representation of the approach to knock-in a C-terminal triple TY-1-P2A-neomycin tag into the human 3’ *BRCA1* locus (top) in RPE1 *TP53*^−/−^ cells and Sanger sequencing results of the region of the integration (bottom). (**B**) Lysates of RPE1 hTERT cell lines either *TP53*^−/−^ or *TP53*^−/−^ *BRCA1-TY-1* endogenously tagged analyzed by western blotting to visualize BRCA1 protein levels. (**C**) Representative images of RPE1 hTERT *TP53*^−/−^ endogenously tagged BRCA1-TY-1 cells IR-treated (10 Gy) and analyzed 4 h post IR to detect TY-1 and RAD51 foci (scale bar 10 µm). (**D**) Volcano plots depicting statistical differences in binding proteins identified by MS proteomic analysis upon TY-1 IP of RPE1 hTERT *TP53*^−/−^ *BRCA1-TY-1* compared to RPE1 hTERT *TP53*^−/−^ cells not irradiated (left) or 1 h post 5 Gy IR (right) (*n* = 3 (biological replicates), fold-changes were determined by Perseus software, two-tiered *t* tests, *P* value < 0.05 was considered significant). Source data are available online for this figure

In a complementary approach, we performed IP-MS on a cell line carrying an endogenous C-terminal triple TY-1 tag on BRCA1 (see Fig. EV2A–C for details on this cell line). In this experiment, we compared the BRCA1-interactome with and without DNA damage induction using ionizing radiation (IR) (Fig. 1G; Dataset EV2). Again, we identified many of the known BRCA1 interactors, and, as has been observed before, these do not show an IR-dependent interaction (Fig. EV2D) (Baer and Ludwig, 2002; Cantor et al, 2001; Greenberg et al, 2006; Manke et al, 2003; Wang et al, 2000; Yu et al, 2003). Interestingly, the strongest IR-dependent interactor, TOPBP1, also appeared in our other dataset as a significant interactor of FL BRCA1, but not BRCA1-∆11 (Fig. 1F,G). Given the previously described observations suggesting that TOPBP1 is involved in HR (Morishima et al, 2007; Moudry et al, 2016; Zhao et al, 2022) and its previously described indirect interaction with BRCA1 via BRIP1 (Greenberg et al, 2006), we decided to follow-up on this exon 11 mediated interaction.

**Figure EV3:**
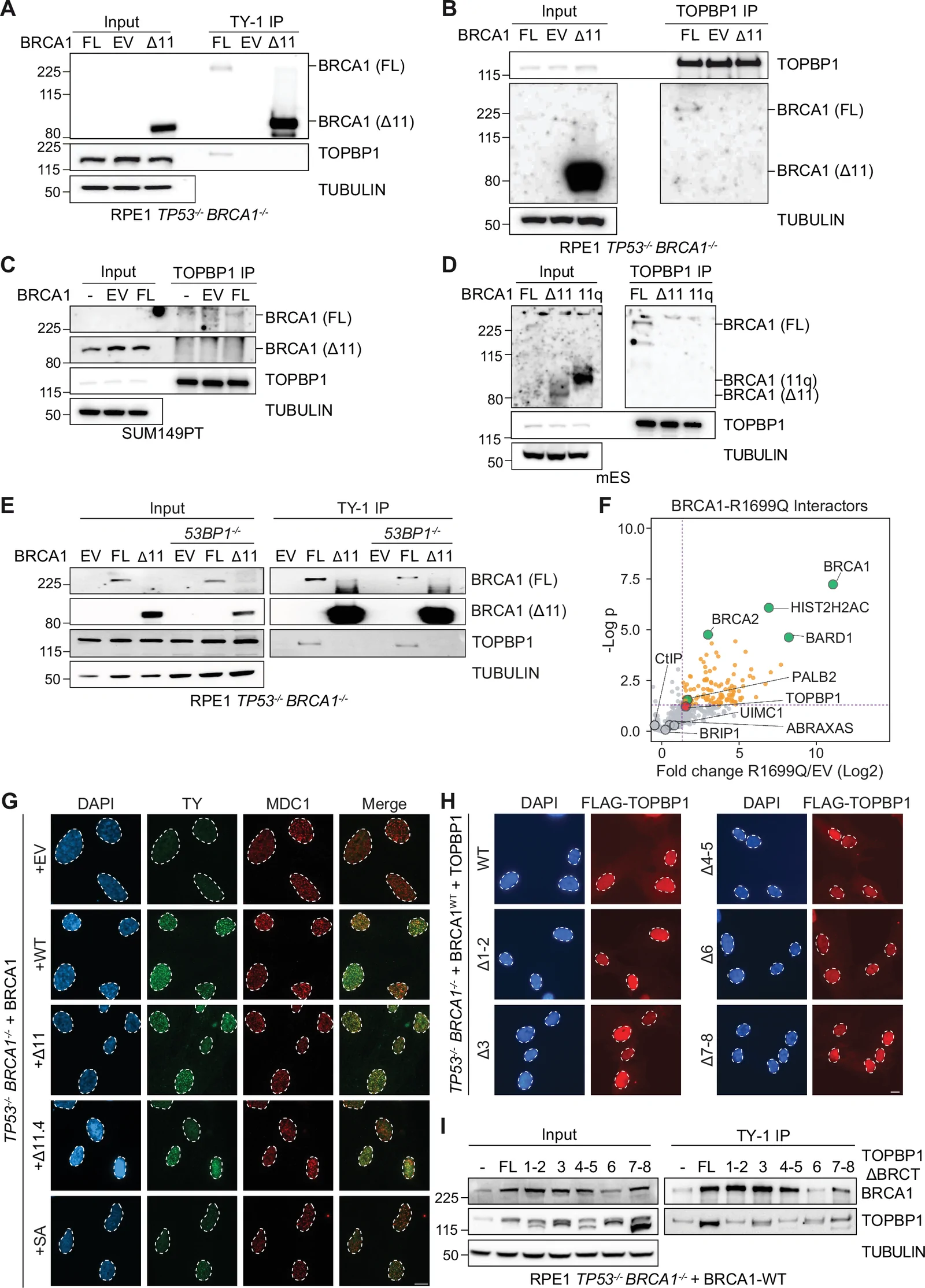
Validation of the interaction between TOPBP1 and BRCA1 exon 11 in different cell systems (related to Figs. 1, 2 and 3). (**A**) Cell lysates harvested 1 h post 5 Gy IR from RPE1 hTERT *TP53*^−/−^ *BRCA1*^−/−^ cells virally reconstituted with dox-inducible TY-1 tagged BRCA1-FL, BRCA1-∆11 or an EV control were subjected to TY-1 IP and analyzed by western blotting. (**B**) Cell lysates harvested 1 h post 5 Gy IR from RPE1 hTERT *TP53*^−/−^ *BRCA1*^−/−^ cells virally reconstituted with dox-inducible TY-1 tagged BRCA1-FL, BRCA1-∆11 or an EV control were subjected to endogenous TOPBP1 IP and analyzed by western blotting. (**C**) SUM149PT cells virally reconstituted with dox-inducible TY-1 tagged BRCA1-FL or EV were subjected to TOPBP1 IP 1 h post 5 Gy IR and analyzed by western blotting. (**D**) Lysates of BRCA1-deficient mES cell lines reconstituted with human BRCA1-FL, -∆11 or -11q were subjected to TOPBP1 IP 1 h post 5 Gy IR and analyzed by western blotting. (**E**) Cell lysates harvested 1 h post 5 Gy IR from RPE1 hTERT *TP53*^−/−^ *BRCA1*^−/−^ or *TP53*^−/−^ *BRCA1*^−/−^ *53BP1*^−/−^ cells virally reconstituted with dox-inducible TY-1 tagged BRCA1-FL, BRCA1-∆11 or an EV control were subjected to TY-1 IP and analyzed by western blotting. (**F**) Volcano plots depicting statistical differences in binding proteins identified by MS proteomic analysis upon TY-1 IP of RPE1 hTERT *TP53*^−/−^ *BRCA1*^−/−^ cells virally reconstituted with dox-inducible BRCA1-R1699Q 1 h post 5 Gy IR compared to EV 1 h post 5 Gy IR (*n* = 3 (biological replicates), fold-changes were determined by Perseus software, two-tiered *t* tests, *P* value < 0.05 was considered significant). (**G**) Representative microscopy images of BRCA1-TY-1 and MDC1 foci from RPE1 hTERT *TP53*^−/−^ *BRCA1*^−/−^ cell lines virally reconstituted with dox-inducible FL BRCA1, BRCA1-∆11, BRCA1-∆11.4, BRCA1-SA or EV control 4 h post 10 Gy (scale bar 10 µm). (**H**) Representative microscopy images of FLAG-stained TOPBP1 nuclear localization from RPE1 hTERT *TP53*^−/−^ *BRCA1*^−/−^ cell lines virally reconstituted with dox-inducible FL BRCA1 and FLAG-tagged FL TOPBP1, TOPBP1-∆1-2, TOPBP1-∆3, TOPBP1-∆4-5, TOPBP1-∆6 or TOPBP1-∆7-8 (scale bar 10 µm). (**I**) Cell lysates harvested 1 h post 5 Gy IR of RPE1 hTERT *TP53*^−/−^ *BRCA1*^−/−^ cells virally reconstituted with dox-inducible BRCA1-FL and the indicated FLAG-tagged TOPBP1 deletion mutants were subjected to TY-1 IP and analyzed by western blotting. Please note that the TOPBP1 staining also visualizes endogenous TOPBP1. Source data are available online for this figure

We confirmed the interaction between exon 11 of BRCA1 and TOPBP1 by performing dedicated IPs in both directions using our virally complemented RPE1 *BRCA1*-KO cells upon IR treatment (Fig. EV3A,B). In addition, we validated the interaction in SUM149PT cells. This cell line, derived from a triple negative breast tumour, contains a frameshifting mutation in exon 11 which results in exon-skipping and expression of a *BRCA1-Δ11q* isoform (Gu et al, 2016). Confirming our data in RPE1 cells, we did not observe an interaction between TOPBP1 and BRCA1-Δ11q upon IR in this more clinically relevant model. As a control, we virally expressed FL BRCA1 in these cells, which restored the interaction with TOPBP1 (Fig. EV3C). We also verified the interaction in mouse embryonic stem (mES) cells. For this, we used a previously described mES cell line carrying a conditional *BRCA1* wild-type allele (Bouwman et al, 2020) and complemented these cells with human FL BRCA1, Δ11 or 11q. Upon depletion of the wild-type mouse allele, the interaction with TOPBP1 was restored only in cells expressing FL BRCA1, and not the variants disrupting exon 11 (Fig. EV3D).

### BRCA1 contains a dual interaction mode for TOPBP1

**Figure 2:**
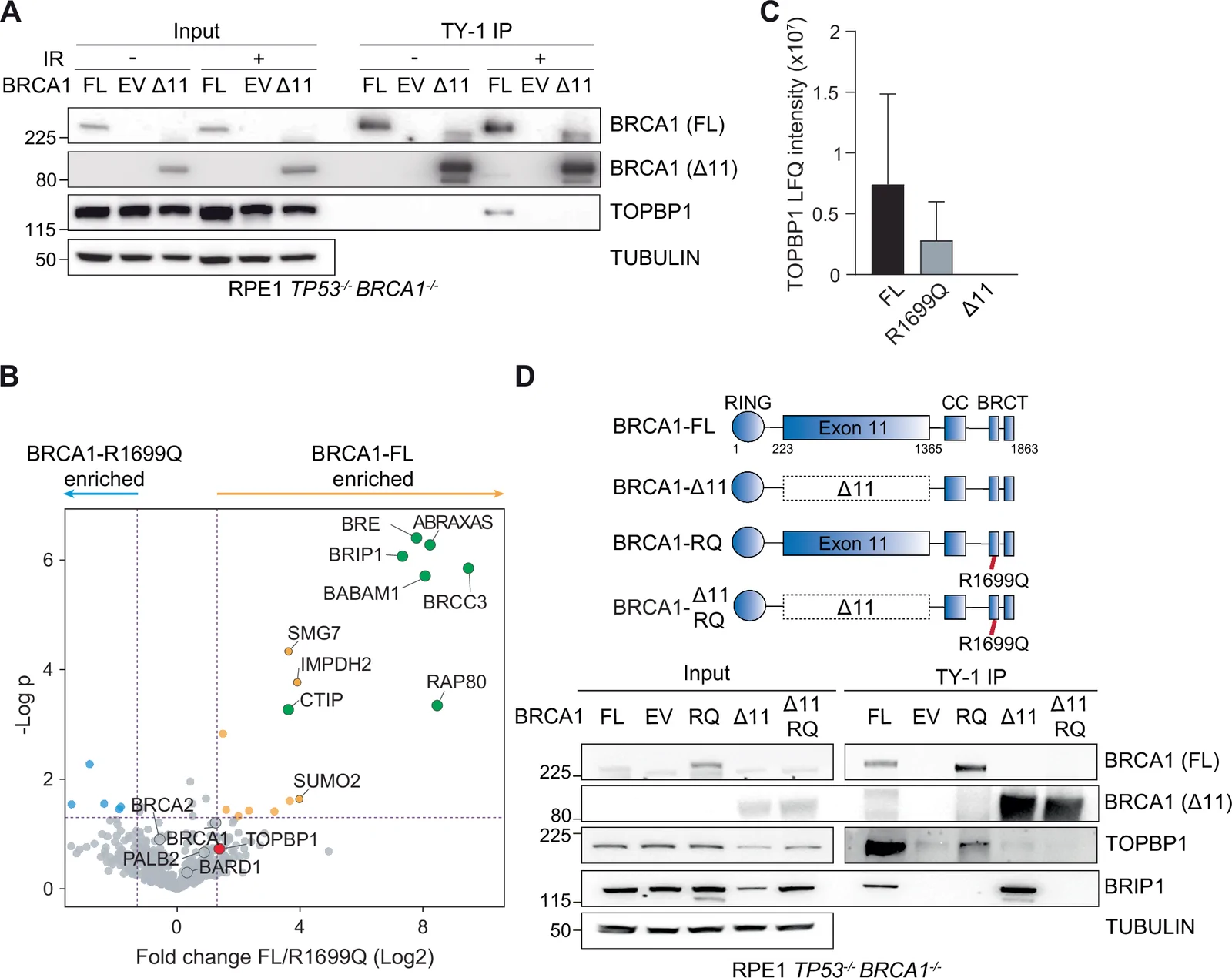
BRCA1 contains a dual interaction mode for TOPBP1. (**A**) Cell lysates from RPE1 hTERT *TP53*^−/−^ *BRCA1*^−/−^ cells virally reconstituted with dox-inducible BRCA1-FL, BRCA1-∆11 or EV control were subjected to TY-1 IP 1 h post 5 Gy IR or noIR and analyzed by western blotting. (**B**) Volcano plot depicting statistical differences in binding proteins identified by MS proteomic analysis upon TY-1 IP of RPE1 hTERT *TP53*^−/−^ *BRCA1*^−/−^ cells virally reconstituted with a dox-inducible BRCA1-FL or BRCA1-R1699Q 1 h post 5 Gy IR (*n* = 3 (biological replicates), fold-changes were determined by Perseus software, two-tiered *t* tests, *P* value < 0.05 was considered significant). (**C**) Graph showing TOPBP1 LFQ intensity from the proteomic analysis of TY-1 IPs 1 h upon 5 Gy IR of RPE1 hTERT cell lines *TP53*^−/−^ *BRCA1*^−/−^ virally reconstituted with BRCA1-FL, BRCA1-R1699Q and BRCA1-∆11 (*n* = 3, mean + SEM (biological replicates)). (**D**) Schematic diagram of BRCA1-FL, BRCA1-∆11, BRCA1 harboring a R1699Q mutation (RQ) or a double ∆11 and R1699Q mutation (∆11, RQ) (left). Lysates of the indicated cell lines were harvested and IP’ed using a TY-1 antibody and analyzed by western blotting 1 h post 5 Gy (right). Source data are available online for this figure.

To confirm the damage-dependency of the interaction between BRCA1 and TOPBP1 in our IP-MS data, we performed a dedicated IP with and without IR in our RPE1 *BRCA1*-KO cells complemented with either FL BRCA1 or BRCA1-Δ11. Confirming our MS results, this IP shows IR-dependent binding of TOPBP1 to FL BRCA1 only, with no binding to BRCA1-Δ11 (Fig. 2A). In the next sections where we have narrowed-down the interaction domains, all IPs were therefore performed after induction of DNA damage by IR. To rule out the possibility that the interaction between BRCA1 and TOPBP1 is driven by active resection and would therefore be reduced by BRCA1-Δ11 expression, we performed our IP experiments in a 53BP1 knock out setting as this has been shown to restore end-resection levels in BRCA1-Δ11 cells (Nacson et al, 2018). Also in this background, we see loss of TOPBP1 binding, indicating the interaction is not purely a consequence of active resection (Fig. EV3E).

Previous research has shown that BRCA1 forms a complex with TOPBP1 via the bridging protein BRIP1 that binds the BRCA1 BRCT domains (Greenberg et al, 2006). To better characterize a possible dual interaction mode between BRCA1 and TOPBP1, we generated cell lines expressing BRCA1 with a mutation in the BRCT domains (R1699Q) in the FL or Δ11 background. We performed IP-MS on the cells expressing FL BRCA1 R1699Q, which confirmed that this mutant disrupts the interaction with ABRAXAS, CtIP and BRIP1 (Figs. 2B and  EV3F; Dataset EV1). Quantification of the TOPBP1 peptides identified in the different MS datasets showed that TOPBP1 was most abundant in the FL BRCA1 IP, followed by the BRCT mutant, whereas in the cells expressing BRCA1-∆11 no peptides corresponding to TOPBP1 were identified at all (Fig. 2C). These data indicate that the interaction between TOPBP1 and BRCA1 is most strongly regulated via exon 11. To confirm the proteomics data, we performed an IP on cells expressing the BRCT mutant either in the FL BRCA1 or ∆11 background. While disruption of either the BRCT or exon 11 reduced binding to TOPBP1, the effect was strongest in cells expressing BRCA1-Δ11 (Fig. 2D). The double mutant did not show any binding to TOPBP1, indeed suggesting a dual binding mode of BRCA1 to TOPBP1. Importantly, the binding of BRCA1 to BRIP1 is functional in ∆11 cells, indicating that deletion of exon 11 does not lead to a disruption of the BRCT-mediated interactions (Figs. 2D,  1F and  EV1C).

Collectively, our findings support an IR-dependent dual-mode interaction between TOPBP1 and BRCA1. A small proportion of BRCA1 is recruited indirectly to TOPBP1 via a BRCT domain-dependent interaction with BRIP1, while the predominant mode of association seems to occur through exon 11. Future biochemical studies will be necessary to determine whether this exon 11-mediated interaction is direct.

### Characterization of the interaction between TOPBP1 and exon 11 of BRCA1

**Figure 3:**
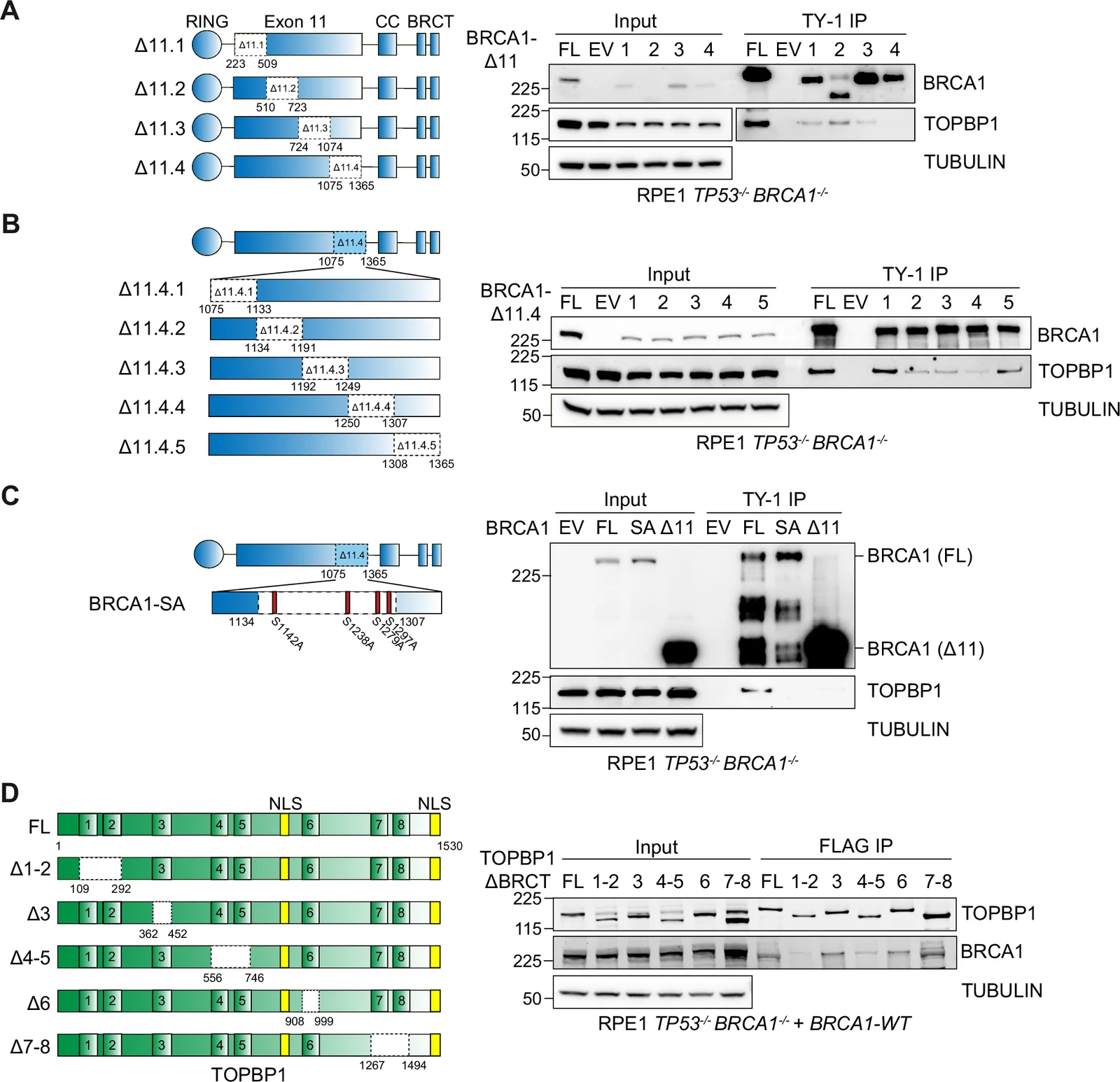
TOPBP1 BRCT1-2 bind to a C-terminal region of BRCA1 exon 11. (**A**) Schematic diagram of BRCA1 exon 11 deletion mutants used in this panel (left). Cell lysates from RPE1 hTERT *TP53*^−/−^ *BRCA1*^−/−^ cells virally reconstituted with dox-inducible BRCA1-FL, the indicated exon 11 deletion mutants or EV control were subjected to TY-1 IP 1 h post 5 Gy IR and analyzed by western blotting (right). (**B**) Schematic diagram of BRCA1 exon 11 C-terminal deletion mutants used in this panel (left). Lysates of the RPE1 hTERT *TP53*^−/−^ *BRCA1*^−/−^ cells virally reconstituted with the indicated dox-inducible TY-1 tagged deletion mutants cells were subjected to TY-1 IP 1 h post 5 Gy IR and analyzed by western blotting (right). (**C**) Schematic diagram of BRCA1-SQ sites in the C-terminus of exon 11 (left). IR-irradiated lysates (1 h post 5 Gy IR) of the indicated cells virally reconstituted with dox-inducible BRCA1-FL or BRCA1-FL in which 4 SQ sites in the C-terminus of exon 11 were mutated to alanines (SA) were immunoprecipitated using a TY-1 antibody and analyzed by western blotting (right). (**D**) Schematic diagram of TOPBP1 single or tandem BRCT deletion mutants used in this study (left). IR-irradiated lysates (1 h post 5 Gy IR) of RPE1 hTERT cell lines *TP53*^−/−^ *BRCA1*^−/−^ virally reconstituted with dox-inducible BRCA1-FL and the indicated flag-tagged TOPBP1 deletion mutants were immunoprecipitated using a FLAG antibody and analyzed by western blotting (right). Source data are available online for this figure.

To further characterize the interaction between BRCA1 and TOPBP1, we generated a series of four smaller deletion mutants in exon 11 (Fig. 3A). In an attempt to minimally affect protein folding, we used prediction software (RaptorX) to prevent disruption of alpha helix and beta sheet structures when creating the deletion constructs (Wang et al, 2016a). IP experiments with these deletion constructs showed reduced TOPBP1 binding to all of them, but only the deletion of the most C-terminal part of exon 11 completely disrupted binding (Fig. 3A). Importantly, this smaller deletion mutant was still recruited to sites of DNA damage (Fig. EV3G). We then further narrowed down the binding interface by creating five deletion constructs of this C-terminal part of exon 11 (Fig. 3B). Although deleting these regions seems to have a small effect on expression levels of BRCA1, deletion of the first or last 58 or 57 amino acids of this fragment did not affect binding, while all three deletion constructs of the middle part showed reduced binding. Together, these data indicate that the binding to TOPBP1 is mediated via a minimal region comprising amino acids 1134–1307.

Previous research has identified an SQ enriched region in BRCA1 (amino acids: 1143–1525) (Traven and Heierhorst, 2005) that partially overlaps with the minimal region we mapped to bind to TOPBP1. Given the DNA damage-dependent nature of the interaction between BRCA1 and TOPBP1, we wondered whether ATM/ATR-dependent phosphorylation of the 4 SQ-sites within this TOPBP1-binding region was required for the binding. To study this, we generated a full length BRCA1 construct where all 4 Serines were mutated to Alanines and tested its interaction to TOPBP1. These mutations impaired binding to TOPBP1 compared to wild-type BRCA1 (Fig. 3C), while DSB recruitment was unaffected by these mutations (Fig. EV3G). These data suggest that damage-dependent phosphorylation of a region in the C-terminus of exon 11 is required for its interaction with TOPBP1. We were unsuccessful to further narrow-down the binding domain, suggesting there might be redundancy between these phospho-sites in regulating binding to TOPBP1.

Vice versa, we also investigated what domain of TOPBP1 directed the binding to BRCA1 exon 11. TOPBP1 consists of a series of eight BRCT domains, known for their interaction to phosphorylated proteins (Day et al, 2021; Leung and Glover, 2011). To study which BRCT domains are involved in the binding to exon 11 of BRCA1, we created cell lines expressing different BRCT deletion constructs of TOPBP1. All deletion constructs were localized in the nucleus, as has been shown before (Cescutti et al, 2010; Yamane et al, 2002) (Fig. EV3H). Immunoprecipitation of the different FLAG-tagged TOPBP1 deletion constructs showed that the constructs lacking the BRCT domains 3, 6 or 7-8 were still able to bind to BRCA1. However, deletion of the first two BRCTs, and to a lesser extent BRCT 4-5, abrogated binding to BRCA1 (Fig. 3D). Performing the IP in the reverse order by isolating BRCA1-TY-1 confirmed these data (Fig. EV3I). A previous study using TOPBP1 point mutations has shown that BRCT1, 5 and 7 are involved in the interaction with BRCA1 (Liu et al, 2017). Moreover, the previously described interaction between BRIP1 and TOPBP1 has been reported to occur via BRCT 7 and 8 of TOPBP1 (Gong et al, 2010). Our data are in line with these previously reported results and indicate that BRCA1 exon 11 binds TOPBP1 via a different region than BRIP1.

### The interaction between TOPBP1 and BRCA1 exon 11 is not involved in checkpoint activation

**Figure EV4:**
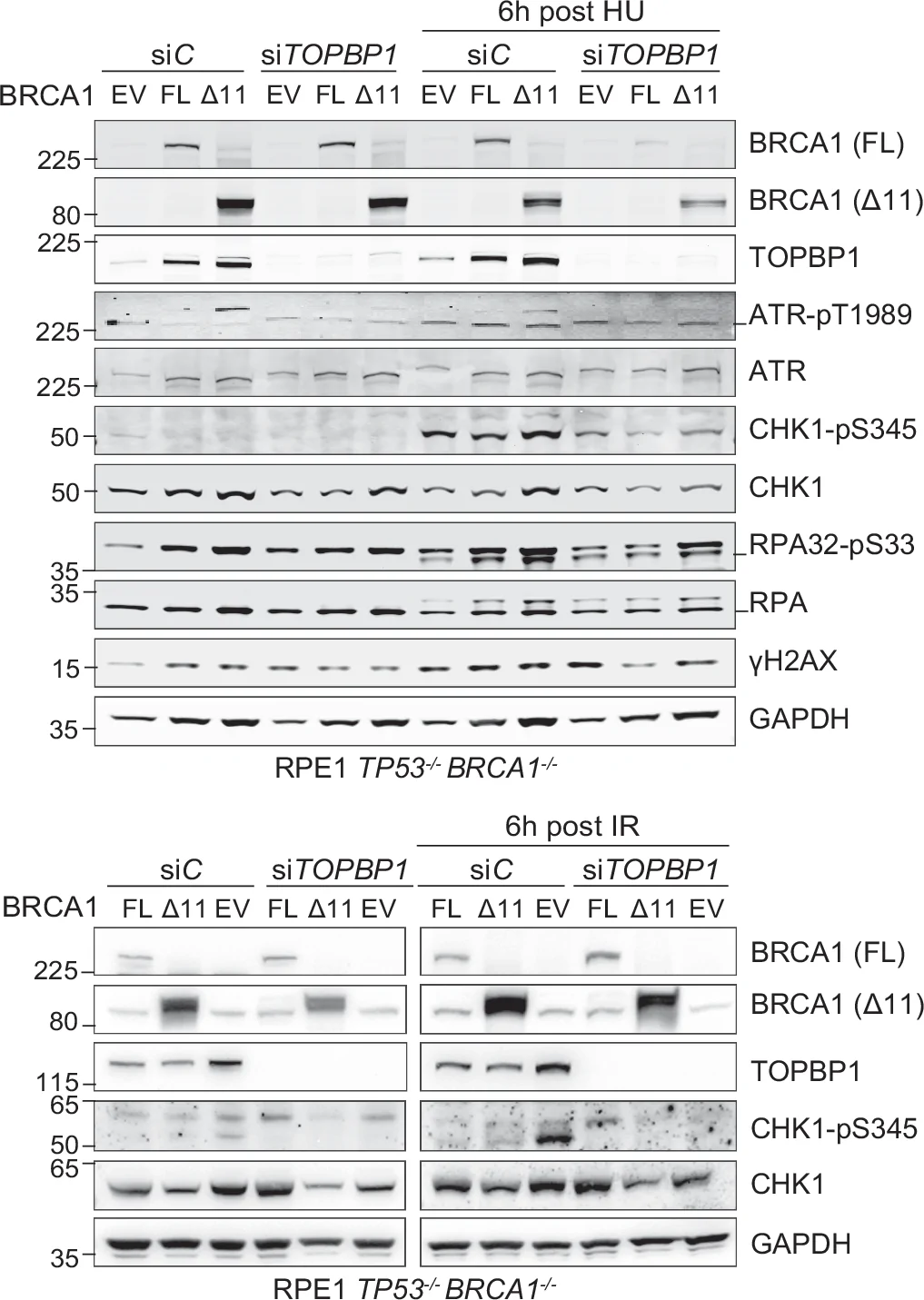
The interaction between BRCA1 exon 11 and TOPBP1 is not required for checkpoint activation (related to Fig. 4). Cell lysates from RPE1 hTERT *TP53*^−/−^ *BRCA1*^−/−^ cells virally reconstituted with dox-inducible BRCA1- FL, BRCA1- ∆11 or EV control and transfected with a control (siC) or *TOPBP1*-targetting siRNA (siTOPBP1) harvested 6 h post 20 mM HU treatment (top) or 6 h post 5 Gy IR (bottom) were analyzed by western blotting using the indicated antibodies. Source data are available online for this figure

Given the well-described role of TOPBP1 in regulating ATR activity and cell cycle checkpoint activation (Furuya et al, 2004; Kumagai et al, 2006; Wardlaw et al, 2014), we investigated whether the interaction with BRCA1 is required for this activity. For this, we analyzed ATR-mediated phosphorylation events by western blot upon hydroxyurea (HU) treatment or IR in cells expressing BRCA1-Δ11 as a read-out for checkpoint activation upon replication stress or DSB induction, respectively. In both conditions, cells expressing FL BRCA1 or BRCA1-Δ11 showed similar ATR activation levels. These data indicate that the interaction between BRCA1 and TOPBP1 is not required for TOPBP1’s function in checkpoint activation (Fig. EV4).

### The BRCA1-TOPBP1 interaction mediates DNA end-resection during DNA repair

**Figure EV5:**
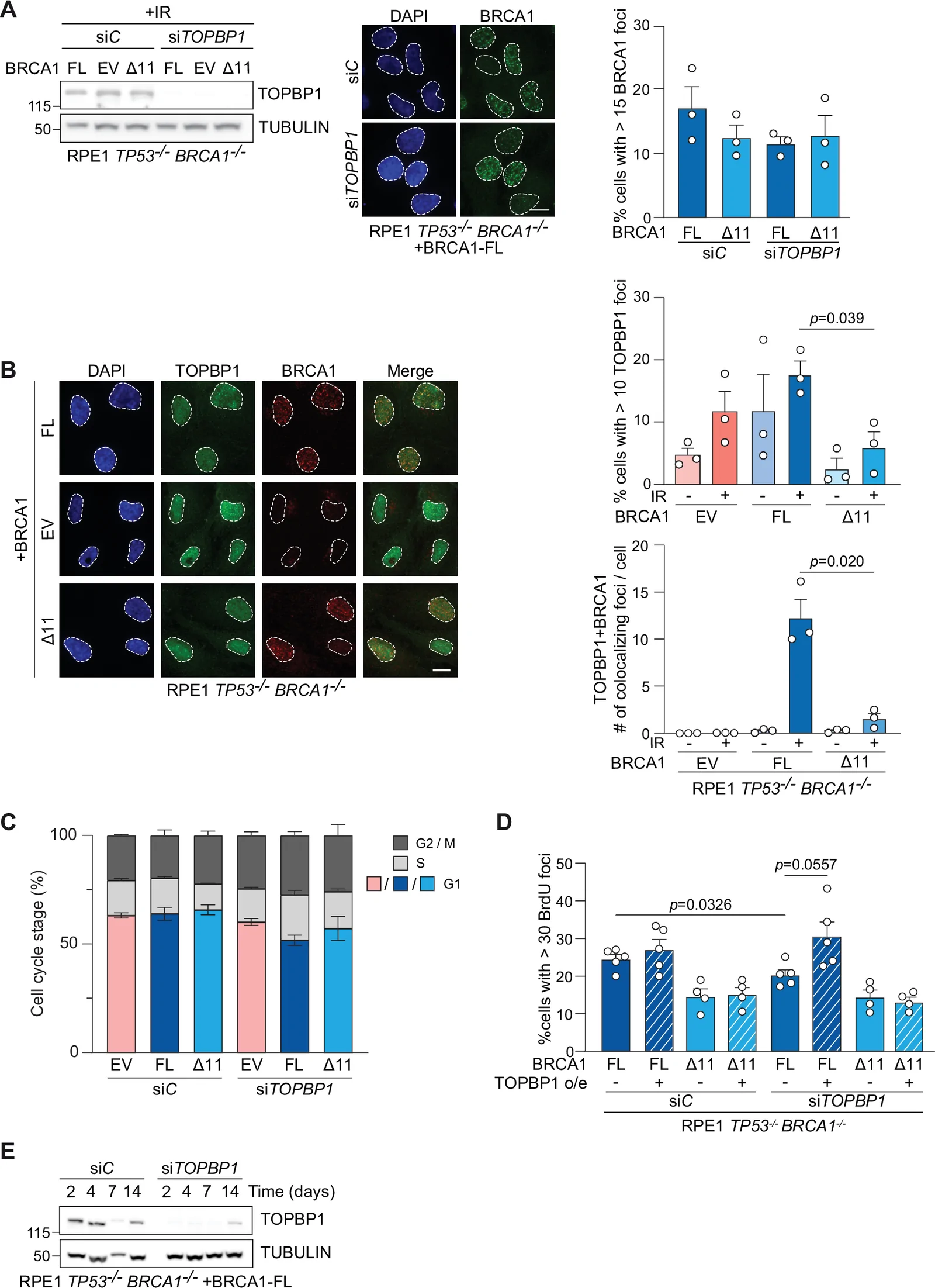
The interaction between BRCA1 and TOPBP1 stimulates DNA end-resection (related to Fig. 4). (**A**) Cell lysates harvested 1 h post 5 Gy IR from RPE1 hTERT *TP53*^−/−^ *BRCA1*^−/−^ cells virally reconstituted with dox-inducible BRCA1-FL, ∆11 or EV control and transfected with a control (siC) or *TOPBP1*-targetting siRNA (siTOPBP1) were analyzed by western blotting to check correct siRNA depletion (left). Representative microscopy images (scale bar 10 µm) (middle) and quantification (right) of BRCA1-TY-1 foci formation 1 h post 5 Gy IR of the same cells (*n* = 3, mean + SEM (biological replicates)). A minimum of 100 cells were counted per replicate. (**B**) Representative microscopy images of TOPBP1 and BRCA1-TY-1 foci from RPE1 hTERT *TP53*^−/−^ *BRCA1*^−/−^ cell lines virally reconstituted with dox-inducible FL BRCA1, an EV control or BRCA1-∆11 1 h post 5 Gy (scale bar 10 µm). Top right graph shows quantification of TOPBP1 foci (*n* = 3, mean + SEM (biological replicates), paired *t* test, *P* values are shown). A minimum of 100 cells were counted per replicate. Bottom right graph shows TOPBP1 + BRCA1 colocalization (*n* = 3, mean + SEM (biological replicates), paired *t* test, *P* values are shown). A minimum of 25 cells were counted per replicate. (**C**) Cell cycle analysis showing percentages of cells in G1, S and G2/M phase as determined by PI staining and flow cytometry (*n* = 3, mean + SEM (biological replicates)). (**D**) Quantification of BrdU foci from cells described in Fig. 4A (*n* = 4 (cells transduced with BRCA1-∆11) or 5 (cells transduced with FL BRCA1), mean + SEM, paired *t* test (biological replicates), *P* values are shown). A minimum of 100 cells were counted per replicate. (**E**) Cell lysates from RPE1 hTERT *TP53*^−/−^ *BRCA1*^−/−^ cells virally reconstituted with TY-1 tagged BRCA1-FL transfected with a control (siC) or *TOPBP1*-targetting siRNA (siTOPBP1) were analyzed by western blotting (related to Fig. 4B). Source data are available online for this figure

To study the biological relevance of the damage-induced interaction of BRCA1 with TOPBP1 in more detail, we set out to investigate whether the disturbed interaction with TOPBP1 is causative for the hypomorphic phenotypes of cells expressing BRCA1-Δ11. Depleting TOPBP1 using siRNAs had a small, non-significant effect on the recruitment of FL BRCA1 to damage sites, similar to the small decrease in recruitment of BRCA1-Δ11 (Figs. EV5A and 1C). Moreover, TOPBP1 depletion did not further reduce recruitment of BRCA1-Δ11 (Fig. EV5A). Vice versa, our data show a clear reduction in TOPBP1 recruitment to sites of DNA damage in cells expressing BRCA1-Δ11 compared to full length BRCA1 (Fig. EV5B). In addition, the majority of the remaining TOPBP1 foci did not colocalize with BRCA1 (Fig. EV5B).

**Figure 4:**
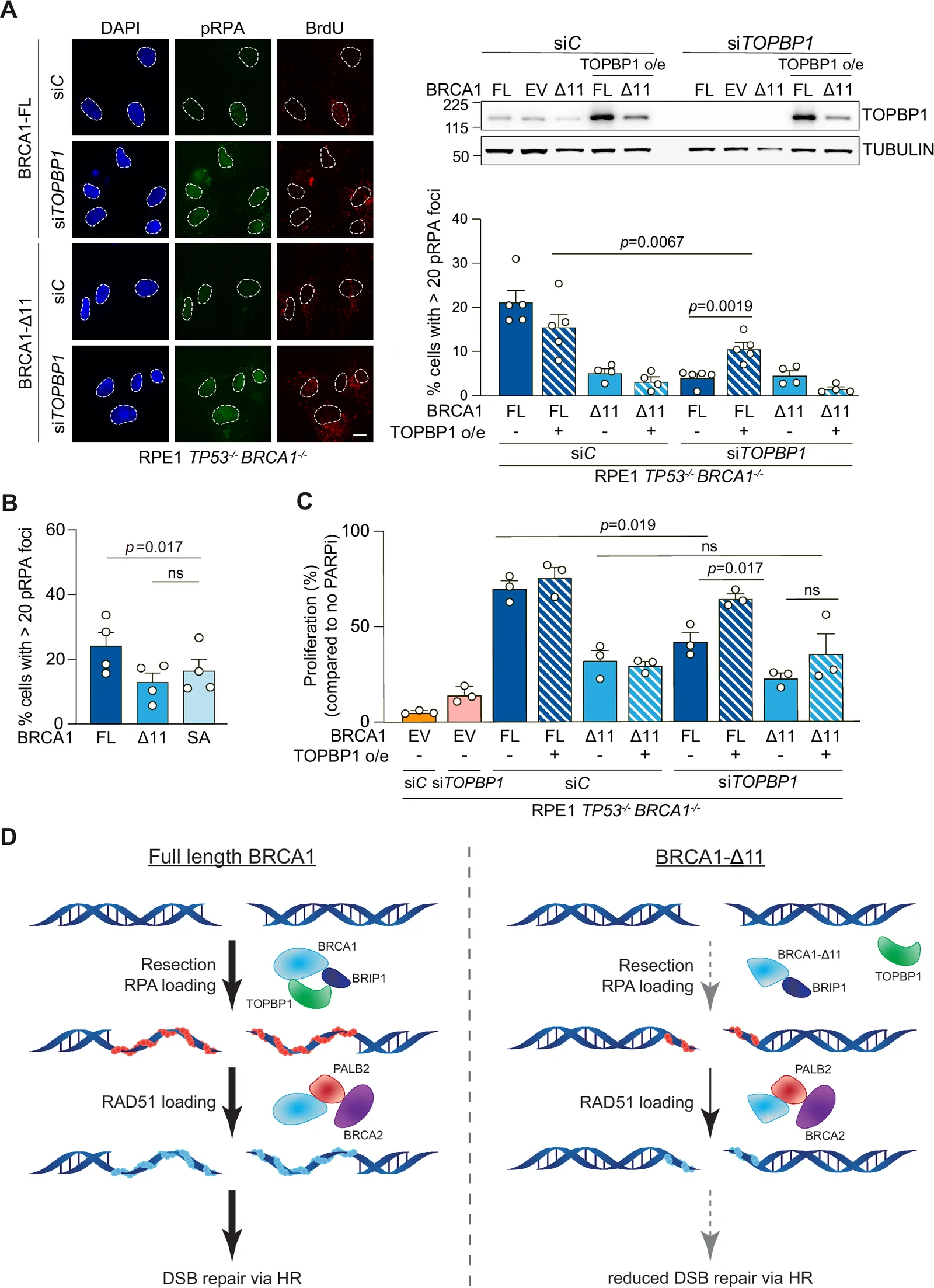
The BRCA1-TOPBP1 complex mediates DNA end-resection during DSB repair. (**A**) RPE1 hTERT *TP53*^−/−^ *BRCA1*^−/−^ cell lines virally reconstituted with dox-inducible FL BRCA1, an EV control or BRCA1-∆11 together with siRNA resistant FL TOPBP1 were transfected with a control (siC) or *TOPBP1*-targetting siRNA (siTOPBP1), followed by IF microscopy to analyze p-RPA (RPA32 pS4/8) and BrdU foci formation 4 h post 10 Gy IR. Left panel shows representative microscopy images of p-RPA and BrdU foci (scale bar 10 µm). Upper right panel shows western blotting analyses of the cells mentioned above and lower right panel shows quantification of p-RPA foci (*n* = 4 (cells transduced with BRCA1-∆11) or 5 (cells transduced with FL BRCA1), mean + SEM (biological replicates), paired *t* test, *P* values are shown). A minimum of 100 cells were counted per replicate. Quantification of BrdU signal is shown in Fig. EV5D. (**B**) *TP53*^−/−^ *BRCA1*^−/−^ virally reconstituted with dox-inducible TY-1 tagged FL BRCA1 wild-type or 4 x SA mutant or BRCA1-∆11 were irradiated with 10 Gy and 4 h post-IR analyzed by IF microscopy to detect pRPA (RPA32 pS4/8) foci formation (*n* = 4, mean + SEM (biological replicates), paired *t* test, *P* values are shown). A minimum of 100 cells were counted per replicate. (**C**) Short-term proliferation assay of the cell lines described in Fig. 4A treated with 1000 nM olaparib and dox. Data is normalized to cells treated with dox, but not olaparib (*n* = 3, mean + SEM (biological replicates), paired *t* test, *P* values are shown). (**D**) Model of the TOPBP1-BRCA1 complex function during homologous recombination. Source data are available online for this figure.

Cells expressing BRCA1-Δ11 show a strong reduction of pRPA foci formation, confirming the resection defect observed by others (Nacson et al, 2020; Nacson et al, 2018; Wang et al, 2016b) (Figs. 1D and 4A). siRNA-mediated depletion of TOPBP1 in cells expressing FL BRCA1 showed a similar reduction in pRPA foci as in cells expressing BRCA1-Δ11 (Fig. 4A), which was not due to a change in cell cycle progression (Fig. EV5C). Moreover, depletion of TOPBP1 did not further decrease pRPA foci formation in cells expressing BRCA1-Δ11, demonstrating an epistatic effect. Ectopic expression of an siRNA-resistant TOPBP1 protein showed a rescue of the end-resection defects in TOPBP1-depleted FL BRCA1 cells. In contrast, this complementation did not show a recovery in BRCA1-Δ11 cells, indicating that the role of exon 11 is not just to regulate TOPBP1 protein levels but there is a mechanistic role for the interaction. Similar results were obtained when analyzing native BrdU staining as an independent read-out of end-resection (Fig. EV5D). To further validate a direct role of the TOPBP1-exon 11 interaction in DNA end-resection, we analysed pRPA foci upon re-expressing full length BRCA1 in which the 4 C-terminal SQ sites in exon 11 were mutated into SA. This mutant showed decreased end-resection with similar levels as BRCA1-Δ11 (Fig. 4B). Altogether, these results show that TOPBP1 is involved in regulating end-resection via its interaction with exon 11 of BRCA1.

To test the impact of the interaction of BRCA1 with TOPBP1 on downstream HR, we studied PARP inhibitor (PARPi) sensitivity in our cell lines. Since *TOPBP1* is an essential gene, we failed in generating clonal knock-out cell lines. Therefore, we depleted TOPBP1 using siRNAs and performed short-term PARPi sensitivity assays by measuring cell proliferation upon 7 days of olaparib treatment. Western blotting confirmed that the used siRNAs against TOPBP1 resulted in TOPBP1 depletion for the duration of this experiment (Fig. EV5E). The depletion of TOPBP1 or the expression of BRCA1-Δ11 resulted in a similar sensitization of cells to PARPi and no additive effect was observed. The increased PARPi sensitivity by depletion of TOPBP1 via siRNAs could be rescued by overexpression of TOPBP1 in cells expressing FL BRCA1, but not BRCA1-Δ11 (Fig. 4C). These results further align with a model in which the complex of BRCA1-TOPBP1 is important for efficient HR (Fig. 4D).

In this manuscript, we provide further understanding of TOPBP1’s role during HR, confirming previous literature showing a role for TOPBP1 in end-resection (Liu et al, 2017; Mooser et al, 2020; Morishima et al, 2007), although others have claimed a resection-independent role of TOPBP1 in HR (Moudry et al, 2016). While TOPBP1 also plays an important role in DSB repair during mitosis (Adam et al, 2021; De Marco Zompit et al, 2022; Leimbacher et al, 2019), this function seems independent of its function with BRCA1 since BRCA1 is not recruited to sites of damage during mitosis (Giunta et al, 2010; Zhang et al, 2011).

In total, our data indicate that the role of exon 11 of BRCA1 in DNA end-resection is mediated via a damage-dependent interaction with TOPBP1 providing future opportunities to further explore the mechanistic role of this BRCA1-TOPBP1 complex during end-resection.

## Methods

**Table:** Reagents and tools table

Reagent/resource	Reference or source	Identifier or catalog number
**Experimental models**		
HEK 293T	ATCC	CRL-3216
RPE1 hTERT	ATCC	CRL-4000
RPE1 hTERT *PAC*^*−/−*^ *TP53*^*−/−*^	This study	N/A
RPE1 hTERT *PAC*^*−/−*^ *TP53*^*−/−*^ *BRCA1*^*−/−*^	This study	N/A
RPE1 hTERT *TP53*^−/−^ *BRCA1*^−/−^	Noordermeer et al, 2018	N/A
RPE1 hTERT *TP53*^−/−^ *BRCA1*^−/−^ *53BP1*^−/−^	Noordermeer et al, 2018	N/A
RPE1 hTERT *PAC*^*−/−*^ *TP53*^*−/−*^ *BRCA1-3xTy-1*	This study	N/A
SUM149PT	Durocher laboratory (Noordermeer et al, 2018)	N/A
mES Brca1^-/loxP^ Pim1^DR-GFP/WT^ 129/Ola E14 IB10	Bouwman et al, 2020	N/A
**Recombinant DNA**		
pLentiCRISPRv2	Addgene	52961 (Sanjana et al, 2014)
YFP-P2A-Neo vector	Stewart-Ornstein and Lahav, 2016	N/A
BRCA1-XhoI-tag-P2A-Neo	This study	N/A
pENTR_1A	Van Attikum Laboratory	N/A
pDONR221	Van Attikum Laboratory	N/A
pCL-MFG-BRCA1	Addgene	12341 (Ruffner and Verma, 1997)
pCW57.1-Puro^res^	Addgene	41393 (Root laboratory)
pCW57.1-BRCA1-3xTY-1 (and deletion mutants)	This study	N/A
pcDNA3-LacR-TopBP1	Addgene	31317 (Lindsey-Boltz and Sancar, 2011)
pCW57.1-Zeocin^res^	This study	N/A
pCW57.1-FLAG-TOPBP1 (and deletion mutants)	This study	N/A
pMDLg/pRRE	Addgene	12251 (Dull et al, 1998)
pRSV-Rev	Addgene	12253 (Dull et al, 1998)
pMD2.G	Addgene	12259 (Dull et al, 1998)
**Antibodies**		
**For WB**		
Rabbit α ATR (1:5000)	Bethyl Laboratories	A300-138A
Rabbit α ATR-pT1989 (1:1000)	Abcam	ab223258
Mouse α BRCA1 (1:1000)	Merck	OP92
Rabbit α BRCA1 (1:1000)	Merck	07-434
Rabbit α BRIP1 (1:2000)	Sigma-Aldrich	B1310-200
Rabbit α CHK1 (1:1000)	Abcam	ab47574
Rabbit α CHK1-pS345 (1:1000)	Cell Signalling Technology	2348S
Rabbit α GAPDH (1:5000)	Sigma-Aldrich	G9545
Mouse α γH2AX (1:1000)	Millipore	05-636
Mouse α RPA32 (1:1000)	Abcam	ab2175
Rabbit α RPA32-pS33 (1:1000)	Bethyl Laboratories	A300-246A
Rabbit α TOPBP1 (1:1000)	Abcam	ab2402
Mouse α TUBULIN (1:5000)	Sigma-Aldrich	T6199
Mouse α TY-1 (1:1000)	Diagenode	C1520054
Goat α Mouse IRDye 800 (1:15,000)	LI-COR	926-68071
Goat α Rabbit IRDye 680 (1:15,000)	LI-COR	926-32210
Goat α Mouse HRP (1:5000)	Thermo Fisher Scientific	31432
Donkey α Rabbit HRP (1:5000)	Thermo Fisher Scientific	31458
**For IF**		
Rabbit α BRCA1 (1:1000)	Millipore	07-434
Mouse α BrdU (1:800)	Amersham	RPN202
Mouse α FLAG (1:2000)	Sigma-Aldrich	F1804
Rabbit α RAD51 (1:15,000)	Bio Academia	70-001
Rabbit α RPA32-pS4/S8 (1:1000)	Bethyl Laboratories	A300-245A
Rabbit α TOPBP1 (1:1000)	Abcam	ab2402
Mouse α TY-1 (1:1000)	Diagenode	C1520054
Goat α Mouse AlexaFluor 488 (1:1000)	Thermo Fisher Scientific	A11029
Goat α Mouse AlexaFluor 555 (1:1000)	Thermo Fisher Scientific	A21424
Goat α Rabbit AlexaFluor 488 (1:1000)	Thermo Fisher Scientific	A11034
Goat α Rabbit AlexaFluor 555 (1:1000)	Thermo Fisher Scientific	A21429
**Oligonucleotides and other sequence-based reagents**		
**gRNAs**		
PAC (KO generation)	ACGCGCGTCGGGCTCGACAT	This study
TP53 (KO generation)	CAGAATGCAAGAAGCCCAGA	This study
BRCA1 (KO generation)	AAGGGTAGCTGTTAGAAGGC	This study
BRCA1 (C-term tagging)	TGGCTGGCTGCAGTCAGTAG	This study
**siRNAs**		
TOPBP1	Ambion/Invitrogen	Cat#: AM16708; Assay ID 108170
**PCR primers for genetic validation of editing**		
*PAC* forward primer (KO generation)	ATGACCGAGTACAAGCCCAC	This study
*PAC* reverse primer (KO generation)	CGGGTCATGCACCAGGTG	This study
*TP53* forward primer (KO generation)	AGGAGAGATGCTGAGGGTGTG	This study
*TP53* reverse primer (KO generation)	TCACTGCCATGGAGGAGC	This study
*BRCA1* forward primer (KO generation)	TCTTTGTGATGTGACACCAGTG	This study
*BRCA1* reverse primer (KO generation)	AAGGACATCACAATAACATCAAGTC	This study
*BRCA1* forward primer (C-term tagging)	CTCCCAAAGTGCTGGGATTA	This study
*BRCA1* reverse primer (C-term tagging)	ACAGAGCAAGACTCCGTCTC	This study
**Essential chemicals, enzymes and other reagents**		
Olaparib	Selleck Chemicals	S1060
**Software**		
ImageJ	https://imagej.net/software/imagej/	
Zen imaging software	Zeiss	
Maxquant	https://maxquant.org	version 2.1.3.0
Perseus	https://maxquant.org/perseus/	version 2.0.7.0
Graphpad Prism	GraphPad	Version 10.5.0

All newly created reagents are available upon request to the corresponding author.

### Cell lines

All cells were kept at 37 °C and 5% CO_2_ levels. BRCA1-deficient cells were kept at low O_2_ levels (3%). RPE1 hTERT and HEK 293T cells were obtained from ATCC (Manassas, VA, USA) and cultured in DMEM high glucose, Glutamax and pyruvate supplemented (Thermo Fisher Scientific, Waltham, MA, USA) with 10% FCS and 1% Penicillin/Streptomycin. RPE1 hTERT *PAC*^*−/−*^ *TP53*^*−/−*^ and RPE1 hTERT *PAC*^*−/−*^ *TP53*^*−/−*^ *BRCA1*^*−/−*^ (referred as p53^−/−^ and *BRCA1*^*−/−*^ in this manuscript) were generated by nucleofection of pLentiCRISPRv2 (Addgene: 52961 (Sanjana et al, 2014)) containing the following sgRNAs sg*PAC*: ACGCGCGTCGGGCTCGACAT; sg*TP53*: CAGAATGCAAGAAGCCCAGA; sg*BRCA1*: AAGGGTAGCTGTTAGAAGGC. Subsequently, cells were clonally expanded and genotyping was performed by PCR amplification and Sanger sequencing of the targeted locus, followed by TIDE analysis (Brinkman et al, 2014). RPE1 hTERT *TP53*^−/−^ *BRCA1*^−/−^ and the isogenic *TP53*^−/−^ *BRCA1*^−/−^ *53BP1*^−/−^ cell lines used in Fig. EV3E have been described before (Noordermeer et al, 2018).

The BRCA1 gene in RPE1 hTERT *PAC*^*−/−*^ *TP53*^*−/−*^ cells was endogenously C-terminally tagged with a triple TY-1 tag using electroporation of the pLentiCRISPR-v2 vector with a BRCA1 sgRNA: 5’-TGGCTGGCTGCAGTCAGTAG-3’ and the C-terminal tagging vector BRCA1-XhoI-tag-P2A-Neo with 500 bps homology arms annealing to BRCA1’s C-terminus, a triple TY-1-tag, P2A and a neomycin resistance cassette derived from the previously described YFP-P2A-Neo vector (Stewart-Ornstein and Lahav, 2016). Subsequently, cells were clonally expanded and genotyping was performed by PCR amplification and Sanger sequencing of the targeted locus.

SUM149PT cells (kind gift from Prof. D. Durocher, Lunenfeld Tanenbaum Research Institute) were cultured in Ham’s F-12 nutrient mix (Thermo Fisher Scientific) supplemented with 5% FCS, 1% of 1 M HEPES, 5 µg/mL insulin (I9278-5ML, Sigma-Aldrich, Burlington, MA, USA), and 1 µg/mL hydrocortisone (H4001-1G, Sigma-Aldrich).

A mouse embryonic stem cell line (mES) was used to express the full-length human *BRCA1* gene and two gene variants with deletions comprising the coding region of exon 11 (∆E11 (r.671_4096del) and ∆E11q (r.788_4096del)) located on a Bacterial Artificial Chromosome (BAC RP11-812O5). The *BRCA1* gene variants were generated using a positive/negative selection procedure in combination with Red/ET BAC recombination as described previously (Hendriks et al, 2014). The three BACs containing either the WT gene or exon 11 deletions were transfected in a hemizygous mouse Brca1 mES line (Brca1^-/loxP^ Pim1^DR-GFP/WT^ 129/Ola E14 IB10) (Bouwman et al, 2020) using 6 μg of BAC DNA to transfect 5 × 10^6^ cells per variant using Lipofectamine 2000 (Invitrogen, Thermo Fisher Scientific). Transfected cells were seeded in 10-cm cell culture dishes and cultured in the presence of G418 (200 μg/ml), starting 24 h post transfection. Thirteen days post transfection cells were treated for 16 h with 1.0 µM 4-Hydroxytamoxifen (4-OHT) to remove the mBrca1 conditional allele. After culturing the mES cells for 6 days in BRL- conditioned mES cell culture medium containing puromycin (1.8 µg/mL), experiments was conducted. Mouse mES cells were cultured in DMEM/F-12 medium (Thermo Fisher Scientific) supplemented with 10% FCS, 1% Penicillin–Streptomycin (5000 U/mL, Thermo Fisher Scientific), 5 mg/mL insulin (Sigma-Aldrich), 5 ng/mL epidermal growth factor (Thermo Fisher Scientific, 53003-018), and 5 ng/mL cholera toxin (Sigma-Aldrich, C8052).

All cells were regularly checked for mycoplasma contamination.

### Plasmids and cloning

Purification of plasmid DNA or PCR products was performed using commercially available kits (Qiagen, Hilden, Germany) according to the manufacturer’s protocol. sgRNAs were cloned into BsmBI-digested pLentiCRISPRv2. To obtain pCW57.1-BRCA1-3xTY-1, full length BRCA1 was PCR-amplified from pCL-MFG-BRCA1 (Addgene, 12341 (Ruffner and Verma, 1997)) with a triple TY-1-tag included in the reverse primer and subsequently cloned into pENTR_1A (Thermo Fisher Scientific) and transferred to pCW57.1 (Addgene, 41393) via gateway cloning. To obtain point mutations in BRCA1, we used site directed mutagenesis in the plasmid pENTR_1A_BRCA1-3xTY-1 followed by gateway reaction to introduce the BRCA1 variant into pCW57.1. To get deletions in BRCA1, PCR and Gibson assembly (E5510S, NEB) was performed in the plasmid pENTR_1A_BRCA1-3xTY-1 followed by gateway cloning into pCW57.1.

To obtain a pCW57.1-FLAG-TOPBP1 plasmid, TOPBP1 CDS was obtained via PCR from the plasmid pcDNA3-LacR-TopBP1 (Addgene, 31317; (Lindsey-Boltz and Sancar, 2011)) and cloned into the plasmid pDONR221. The resultant plasmid was subjected to site directed PCR mutagenesis to create silent mutations at the genomic locus of the gRNA used for the depletion. Finally, a gateway reaction was performed to introduce the TOPBP1 cassette into pCW57.1-Zeo. pCW57.1-Zeo was created by exchanging the puro resistance cassette with a Zeocin cassette into the plasmid pCW57.1 (using restriction digestion with AscI and NheI). To create TOPBP1 deletion variants, PCR and subsequent Gibson assembly was performed in the plasmid pCW57.1-FLAG-TOPBP1.

### Viral transductions and transfections

Lentivirus were produced in HEK 293 T cells by jetPEI transfection (Polyplus Transfection, Illkirch, France) of pLentiCRISPRv2 or pCW57.1 plasmids with third generation packaging vectors pMDLg/pRRE, pRSV-Rev and pMD2.G (Addgene, 12251, 12253, 12259 (Dull et al, 1998)). Viral supernatants were harvested 48–72 h post transfection and used to transduce cells at an MOI of ~1 in the presence of 4 µg/mL polybrene. For RPE1 cells with an intact PAC gene, 10 µg/mL puromycin was used for selection 48 h post-transduction, for RPE1 *PAC*^*−/−*^ cells, 1–2 µg/mL of puromycin was used. For zeocin selection, 200 µg/mL were used. Transfections with siRNAs were performed using Lipofectamine RNAiMAX (Invitrogen, Carlsbad, CA, USA) according to the manufacturer’s protocol. Transfected cells were used for experiments 48–72 h after transfection.

### Western blotting

Cells were lysed in RIPA lysis buffer (1% NP-40, 50 mM Tris-HCl pH 7.5, 150 mM NaCl, 0.1% SDS, 3 mM MgCl_2_, 0.5% sodium deoxycholate) supplemented with cOmplete Protease Inhibitor Cocktail EDTA-free (Sigma-Aldrich) and 100 U/mL Benzonase (Sigma-Aldrich). In total, 4× BOLT LDS sample buffer (Thermo Fisher Scientific) with 10× BOLT sample reducing agent containing DTT (Thermo Fisher Scientific) was added to the lysates to obtain 1× dilutions, followed by denaturation at 95 °C for 5 min. Proteins were separated by SDS-PAGE on 4–12% gradient gels (Thermo Fisher Scientific) and transferred to Amersham Protran premium 0.45 µm nitrocellulose membrane (GE Healthcare Life Sciences, Chicago, IL, USA). Membranes were blocked with 8% skimmed milk (Santa Cruz Biotechnology, Dallas, TX, USA) or with Blocking buffer for fluorescent western blot (Rockland, Pottstown, PA, USA), and stained with primary and secondary antibodies in the same buffer used for blocking. After secondary antibody staining, membranes were imaged on an Odyssey CLx scanner (LI-COR BioSciences, Milton, UK), followed by image analysis using ImageStudio (LI-COR BioSciences). Alternatively, when HRP-labelled secondary antibodies were used, the membranes were treated with the WesternBright ECL HRP Substrate kit (Advansta, San Jose, CA, USA) and imaged on an Amersham Imager 680 (Bioké, Leiden, The Netherlands) or iBright (Thermo Fisher Scientific).

### Immunoprecipitation

Cells were grown in 2 or 3 15-cm dishes up to 90% confluency. Twenty-four hours after seeding cells were treated with dox for 48 h. Cells were collected 1 h post IR (5 Gy) in 1 mL ice-cold NETT buffer (100 mM NaCl, 50 mM TRIS pH 7.6, 5 mM EDTA pH 8.0) supplemented with 1× cOmplete protease inhibitor cocktail EDTA-free (Sigma-Aldrich), 0.5% Triton X-100, 7 mM MgCl_2_ and 500 U of benzonase (EMD Millipore). After cell lysis and clearance by centrifugation (14,000 rpm, 10 min), supernatants were incubated for 4 h with 20 μL Dynabeads protein-G (Thermo Fisher Scientific) that were pre-incubated for 2 h and 30 min with 2.2 μg α-TY-1 antibody (Diagenode; C1520054) to immunoprecipitate BRCA1. To immunoprecipitate TOPBP1, lysates were incubated with 2 μg α-TOPBP1 (Abcam; ab2402) and 20 μL Dynabeads protein-A (Thermo Fisher Scientific). For FLAG-IP, lysates were incubated with 2 μg α-FLAG (Sigma-Aldrich; F1804) and 20 μL Dynabeads protein-G.

In the case of subsequent SDS-PAGE and western blotting, lysates were washes five times with NETT buffer and denatured with 2× Bolt LDS sample buffer + reducing agent and incubated at 95 °C for 5 min before loading.

### LC-MS/MS

#### On-bead digestion

After TY-1 immunoprecipitation, samples were washed 3 times with NETT buffer and four times with 50 mM ammonium bicarbonate (ABC) prepared in HPLC grade (keratin free) water. For reduction and alkylation of the samples, 10 mM DTT (prepared in 50 mM ABC) was added to the beads and incubated 25 min at 60 °C. Next, 15 mM 2-Chloroacetamide (2CAA) (prepared in 50 mM ABC) was added to the beads and incubated 25 min at room temperature in the dark. Afterwards, 0.8 µl of 500 mM DTT was added to neutralise the 2CAA and incubated 5 min at room temperature.

For the trypsin digestion, 160 µl 50 mM ABC and 1 μL Trypsin (1 mg/mL stock) was added to the beads and incubated overnight at 37 °C while nutating. Afterwards, 0.5 μL Trypsin was added and incubated for an additional 3 h at 37 °C while nutating. Beads were washed two times with 100 μL ABC, and supernatants were pooled to a new tube, followed by a centrifugation at 10,000 × *g*. Samples were dried in a speed vacuum and stored at −20 °C.

#### Stage tip clean-up

Stagetips (C18, SP-301, Thermo Fisher Scientific) were prewetted with 50 μl 100% Methanol and centrifuged 2 min at 70×* g*. In total, 50 μL of buffer B (80% Acetonitrile, 1% TFA, 0.5% Acetic Acid) was added to each stagetip and centrifuged 2 min at 70 × *g*. In all, 50 μl of buffer A (1% TFA, 0.5% Acetic Acid in HPLC (keratin-free) water) was added and centrifuged 2 min at 70 × *g*. After preparation of the stagetips, freeze-dried peptides were dissolved in 50 μl 1% TFA and centrifuged at maximum speed for 10 min. Afterwards, dissolved peptides were added to the equilibrated stagetips and passed by low-speed centrifugation (5 min at 100 × *g*). Next, stagetips were washed with 50 μl of buffer A and centrifuged at 15 × *g* per 5 min. Peptides were eluted using 20 μl of buffer B and centrifuged 2 min at 10 × *g*. These last two steps were repeated and next, peptides were speed vacuumed until complete dryness and stored for mass spectrometry.

#### Mass spectrometry

Peptides were dissolved in 0.1% formic acid and subsequently analyzed by online C18 nano-HPLC MS/MS with a system consisting of an Easy nLC 1200 gradient HPLC system (Thermo Fisher Scientific), and an Orbitrap Fusion LUMOS mass spectrometer (Thermo Fisher Scientific). Fractions were injected onto a homemade precolumn (100 μm × 15 mm; Reprosil-Pur C18-AQ 3 μm, Dr Maisch, Ammerbuch, Germany) and eluted via a homemade analytical nano-HPLC column (30 cm × 75 μm; Reprosil-Pur C18-AQ 1.9 μm). The analytical column temperature was maintained at 50 °C with a PRSO-V2 column oven (Sonation, Biberach, Germany). The gradient was run from 2 to 36% solvent B (20/80/0.1 water/acetonitrile/formic acid (FA) v/v) in 120 min. Solvent A was water/formic acid 100/0.1 (v/v). The nano-HPLC column was drawn to a tip of ~5 μm and acted as the electrospray needle of the MS source.

The mass spectrometer was operated in data-dependent MS/MS mode with a cycle time of 3 s, with the HCD collision energy at 32% and recording of the MS2 spectrum in the Orbitrap, with a quadrupole isolation width of 1.2 Da. In the master scan (MS1) the resolution was 120,000, the scan range 400–1500, at standard AGC target at maximum fill time of 50 ms. A lock mass correction on the background ion *m/z* = 445.12003 was used. Precursors were dynamically excluded after *n* = 1 with an exclusion duration of 45 s, and with a precursor range of 20 ppm. Charge states 2–5 were included. For MS2 the first mass was set to 110 Da, and the MS2 scan resolution was 30,000 at an AGC target “standard” at maximum fill time of 60 ms.

### Mass spectrometry data analysis

All raw data were analyzed using MaxQuant (version 2.1.3.0) as previously described (Tyanova et al, 2016a). We performed the search against an in silico digested UniProt reference proteome for Homo sapiens including canonical and isoform sequences (January 24, 2022). Database searches were performed according to standard settings after reduction and alkylation of samples: Oxidation (M) and acetyl (Protein N-term) were allowed as variable modifications while Carbamidomethyl (C) was allowed as fixed modification, keeping the maximum number of modifications per peptide equal to 3. Digestion was performed with Trypsin/P, allowing a maximum of 4 missed cleavages. Label-Free Quantification (LFQ) was enabled, not allowing Fast LFQ while permitting iBAQ and matching between runs.

Output from MaxQuant Data was exported and processed for statistical analysis in the Perseus computational platform version 2.0.7.0 (Tyanova et al, 2016b). LFQ intensity values were log2 transformed and potential contaminants and proteins either identified by site or only reverse peptides were removed. Samples were grouped in experimental categories and the matrix was separated in two for further filtering. For the exogenous BRCA1-3xTY-1 samples, proteins not identified in three replicates in at least one group were removed. For the endogenous BRCA1-TY-1 IP runs, proteins not identified in two out of three replicates in at least one group were removed. Missing values were imputed using normally distributed values with 0.3 width and 1.8 down shift for the total matrix. After imputation, Student’s *t* tests with a threshold *P* value of 0.05 and S0 = 1 for both sides were applied. Statistical analysis tables were exported and processed in MS Excel. Volcano plots were constructed using VolcaNoseR (Goedhart and Luijsterburg, 2020) web app with a significance threshold of 1.3 (*P* < 0.05).

### Immunofluorescence

Cells were grown with dox in 24-well format containing 13 mm glass coverslips up to 85% confluency, treated with Dox for 48 h if required, and irradiated with 10 Gy to study IR-induced foci. For p-RPA and BrdU foci analyses, cells were pre-extracted after 4 h post-IR with cold nuclear extraction (NuEx) buffer (20 mM HEPES pH 7.5, 20 mM NaCl, 5 mM MgCl_2_ supplemented with 1 mM DTT, 0.5% NP-40 (Sigma-Aldrich), 1× cOmplete protease inhibitor cocktail (Sigma-Aldrich)) for 12 min at 4 °C and fixed with 4% paraformaldehyde in PBS for 20 min at room temperature and gentle shaking.

For RAD51, TOPBP1 and BRCA1 foci observation, 4 h after irradiation, cells were fixed and permeabilized with 1% paraformaldehyde, 0.3% Triton-X100 in PBS for 20 min at room temperature, follow by a second fixation step with 1% paraformaldehyde, 0.3% Triton-X100 and 0.5% methanol in PBS for 20 min at room temperature.

For TOPBP1 nuclear localization cells were fixed and permeabilized with 2% paraformaldehyde, 0.3% Triton-X100 in PBS for 20 min at room temperature. For MDC1 and TY foci colocalization, 4 h after irradiation, cells were fixed with ice-cold methanol for 30 min at −20 °C.

After fixation, cells were washed three times with PBS and blocked with PBS^+^ (5 g/L BSA, 1.5 g/L glycine in PBS 1×). Antibody dilutions were made in PBS^+^ and incubated at room temperature. After 5 washes with PBS, coverslips were mounted using Aqua Poly/Mount (Polysciences, Warrington, USA). Images were taken using Zeiss Axio Imager 2 fluorescent microscope with a ×40 zoom. Foci of at least 100 cells per condition per replicate were quantified using the IRIF analysis 3.2 Plugin in ImageJ (Luijsterburg et al, 2016).

### Clonogenic survival assay

RPE1 hTERT cells were seeded in 10-cm dishes (RPE1 WT or RPE1 BRCA1^−/−^ complemented with BRCA1-WT: 250 cells; RPE1 BRCA1^−/−^ or complemented with EV: 1500 cells, RPE1 BRCA1^−/−^ complemented with BRCA1-∆11: 500 cells) and treated as indicated. Medium containing olaparib (16 nM) (Selleck Chemicals, Planegg, Germany) and dox (1 µg/mL) was refreshed after 7 days. After 14 days, colonies were stained with crystal violet (0.4% (w/v) crystal violet, 20% methanol) and counted manually.

### Short-term proliferation assays

Cells were seeded into six-well format in the presence of dox and olaparib (1000 nM) in complete culture medium (DMEM + 10% FCS + Pen/Strep) in duplicate. Cells were transfected with siControl (Thermo Fisher Scientific, Invitrogen; 4390843) or siTOPBP1 (Thermo Fisher Scientific, Invitrogen; Assay ID 108170) and after 7 days, cells were washed with PBS, trypsinised and collected. Viable cells were counted using a Vi-CELL XR Cell Viability Analyzer (Beckman Coulter).

### Flow cytometry for cell cycle determination

Cells were grown in six-well format. Twenty-four hours after seeding cells were treated with dox for 48 h. Cells were transfected with siControl (Thermo Fisher Scientific, Invitrogen; 4390843) or siTOPBP1 (Thermo Fisher Scientific, Invitrogen; Assay ID 108170). Cells were then harvested and washed with PBS by centrifugation and fixed in 70% Ethanol. After fixation cells were incubated for 30 min in 0.5 mL PI staining buffer (20 µg/mL Propidium Iodide (VWR), 50 µg/mL RNaseA (Sigma-Aldrich), 0.02% Triton X100 in PBS) after which cell cycle was measured using a NovoCyte 2000 Flow Cytometer (Acea Biosciences) and analysed using NovoExpress Software (Acea Biosciences).

### Statistical analysis

Statistical tests used for data analysis are indicated in the figure legends, including details on biological replicates. Graphpad and Perseus software were used for statistical analysis. No sample size calculations were performed prior to experimental setup, nor were data analyzed blinded.

## Supplementary information

Peer Review File

Dataset EV1

Dataset EV2

Source data Fig. 1

Source data Fig. 2

Source data Fig. 3

Source data Fig. 4

Figure EV1 Source Data

Figure EV2 Source Data

Figure EV3 Source Data

Figure EV4 Source Data

Figure EV5 Source Data

Expanded View Figures

## Data Availability

The full proteomic datasets are uploaded to ProteomeXchange with dataset identifier PXD079087 (https://www.ebi.ac.uk/pride/archive/projects/PXD079087) and raw microscopy data can be found at Mendeley data (https://doi.org/10.17632/3m7552nbs7.1). The source data of this paper are collected in the following database record: biostudies:S-SCDT-10_1038-S44319-026-00904-3.
