# Supplementary material for: DNA end-resection is stimulated by an interaction between BRCA1 exon 11 and TOPBP1
Source: EMBO Rep. 2026 Aug 27;27(19):5468–89. doi: 10.1038/s44319-026-00904-3 (PMC13650151; doi:10.1038/s44319-026-00904-3)
Supplement: Supplementary file 1 [file 44319_2026_904_MOESM13_ESM.pdf]

Expanded View Figures

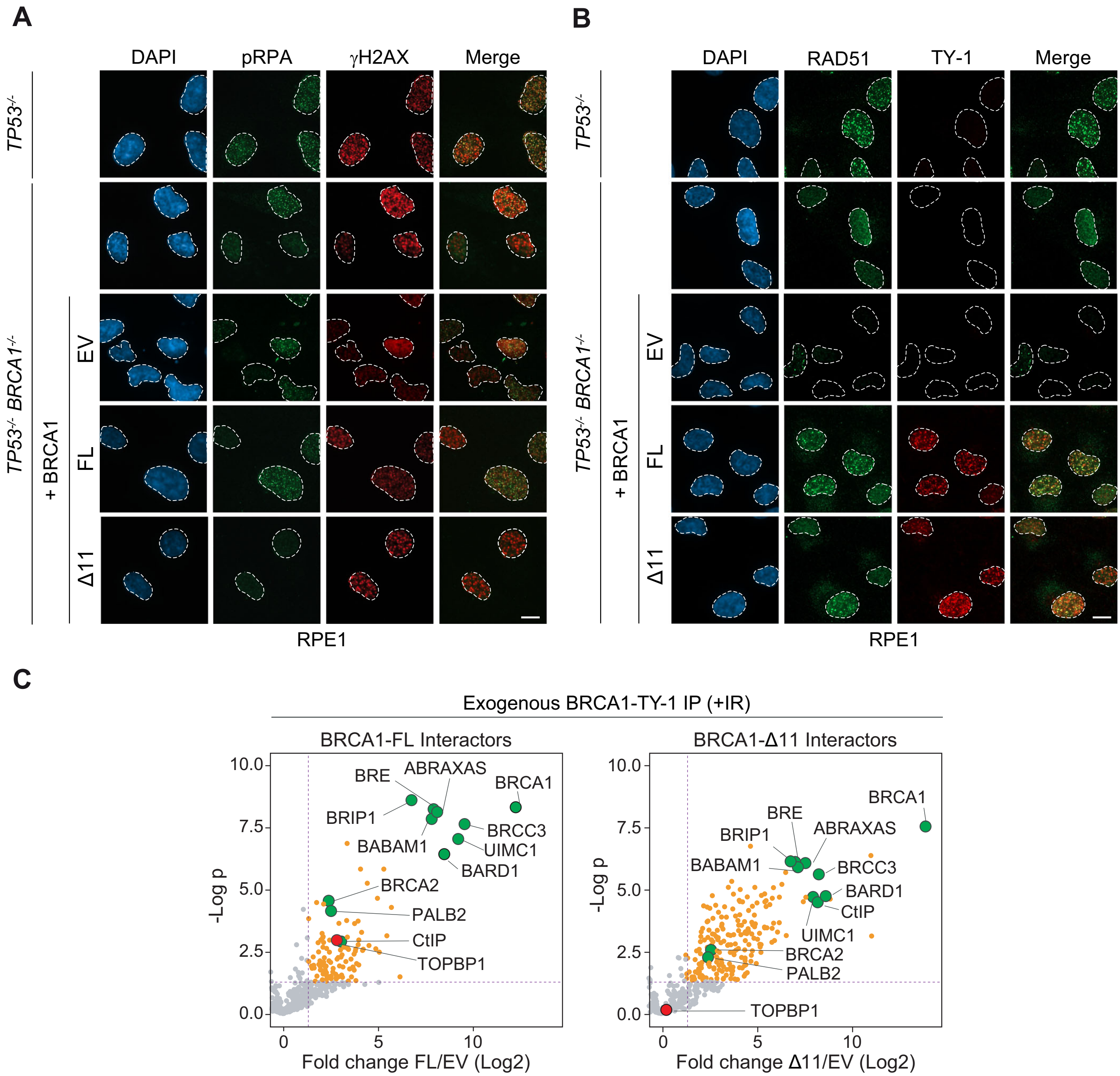

Figure EV1. BRCA1 exon 11 mediates HR and interacts directly to TOPBP1 (related to Fig. 1).

Representative microscopy images of p-RPA (**A**) or RAD51 foci (**B**) quantified in Fig. 1B,C, respectively (scale bar 10 µm). (**C**) Volcano plots depicting statistical differences in binding proteins identified by MS proteomic analysis upon TY-1 IP of RPE1 hTERT *TP53*$^{-/-}$ *BRCA1*$^{-/-}$ cells virally reconstituted with dox-inducible BRCA1-FL or BRCA1-Δ11 1 h post 5 Gy IR compared to EV 1 h post 5 Gy IR ($n = 3$ (biological replicates), fold-changes were determined by Perseus software, two-tiered t tests, P value < 0.05 was considered significant). Source data are available online for this figure

 © The Author(s)

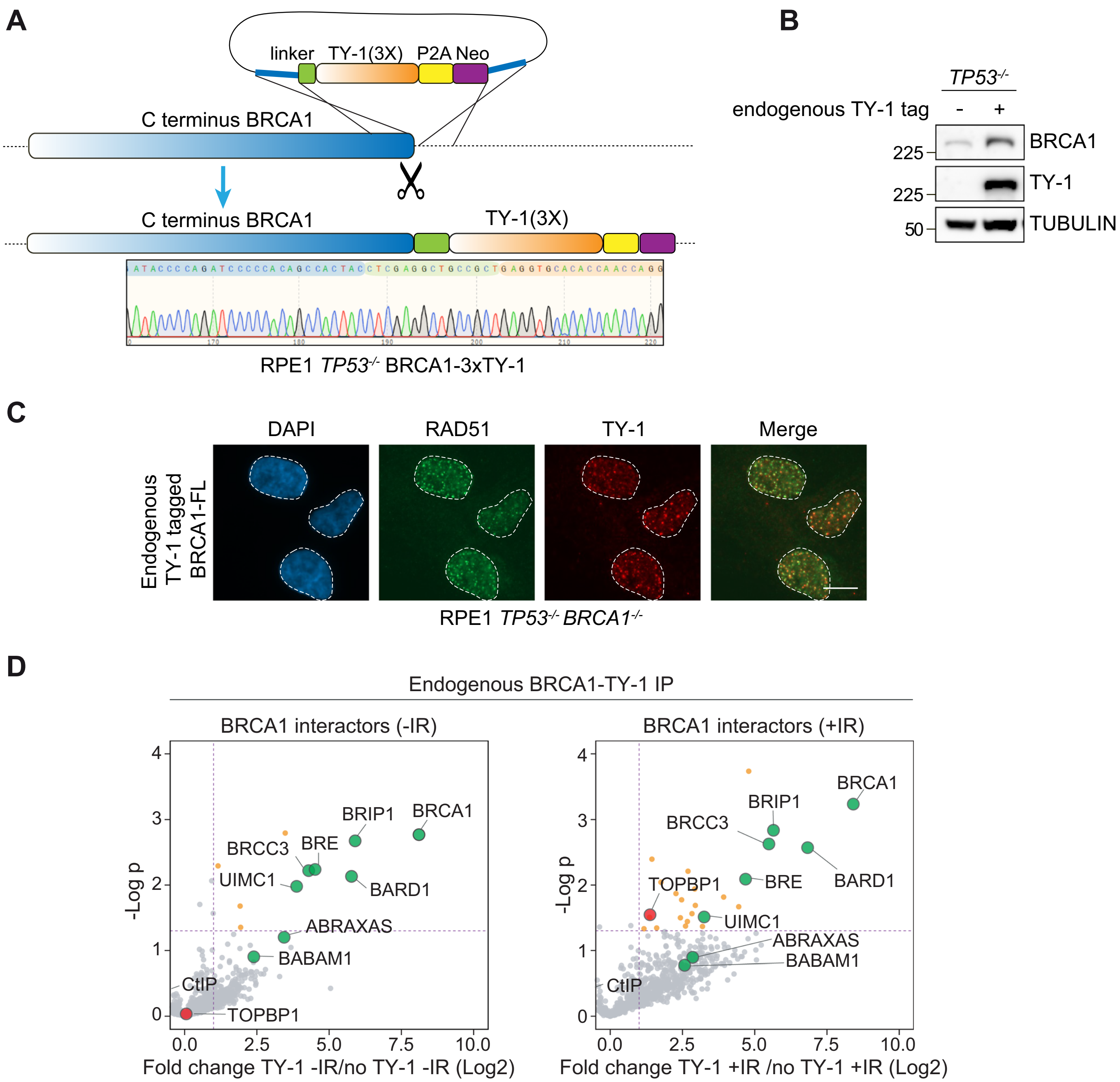

Figure EV2. Generation of an endogenous BRCA1-TY-1 cell line and analyses of its interactors by mass spectrometry (related to Fig. 1).

(**A**) Schematic representation of the approach to knock-in a C-terminal triple TY-1-P2A-neomycin tag into the human 3′ *BRCA1* locus (top) in RPE1 *TP53*$^{-/-}$ cells and Sanger sequencing results of the region of the integration (bottom). (**B**) Lysates of RPE1 hTERT cell lines either *TP53*$^{-/-}$ or *TP53*$^{-/-}$ *BRCA1-TY-1* endogenously tagged analyzed by western blotting to visualize BRCA1 protein levels. (**C**) Representative images of RPE1 hTERT *TP53*$^{-/-}$ endogenously tagged BRCA1-TY-1 cells IR-treated (10 Gy) and analyzed 4 h post IR to detect TY-1 and RAD51 foci (scale bar 10 μm). (**D**) Volcano plots depicting statistical differences in binding proteins identified by MS proteomic analysis upon TY-1 IP of RPE1 hTERT *TP53*$^{-/-}$ *BRCA1-TY-1* compared to RPE1 hTERT *TP53*$^{-/-}$ cells not irradiated (left) or 1 h post 5 Gy IR (right) ($n = 3$ (biological replicates), fold-changes were determined by Perseus software, two-tiered *t* tests, *P* value < 0.05 was considered significant). Source data are available online for this figure

© The Author(s)

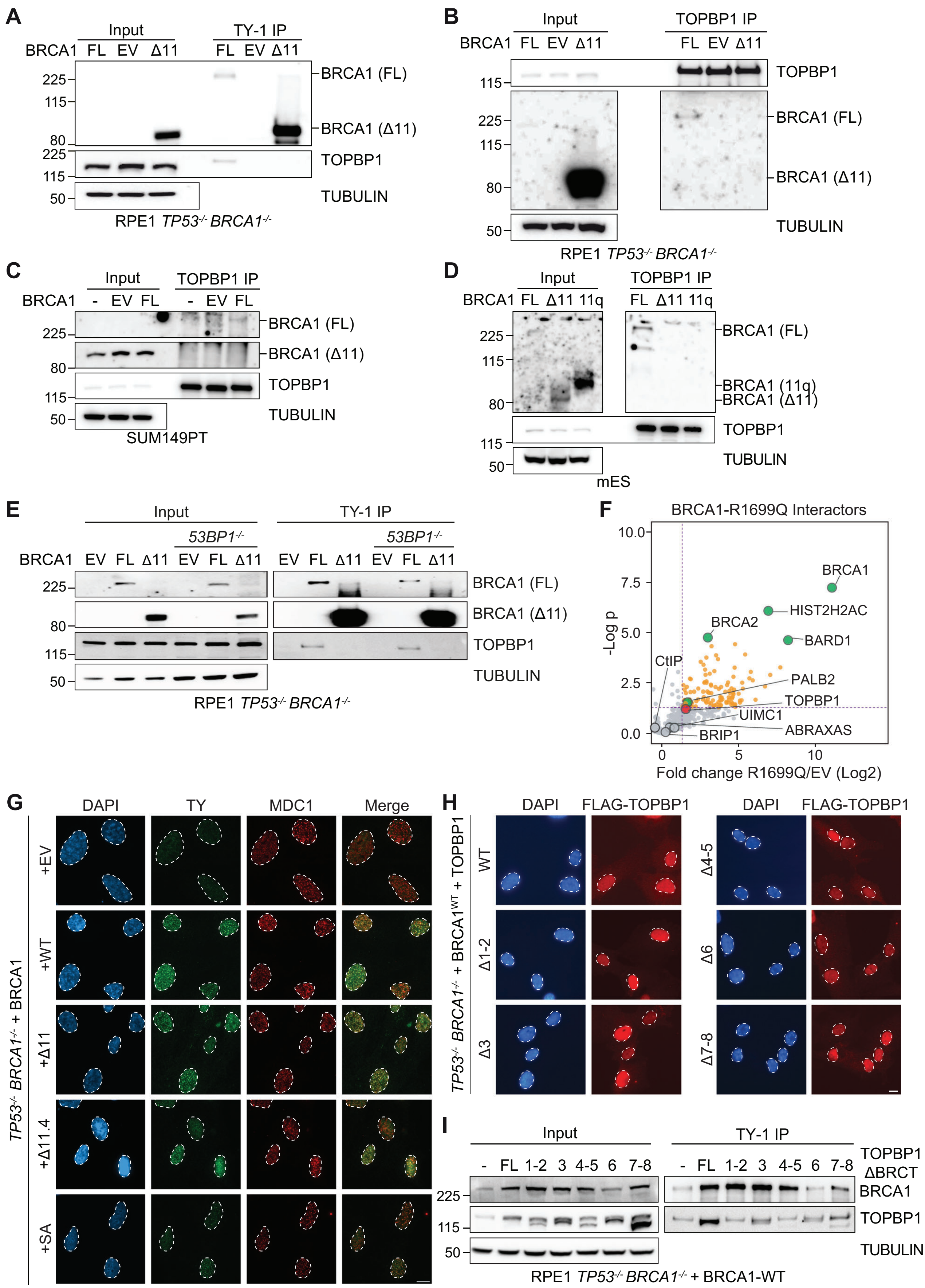
A
Input
TY-1 IP
BRCA1 FL EV Δ11 FL EV Δ11
225
115
80
225
115
50
BRCA1 (FL)
BRCA1 (Δ11)
TOPBP1
TUBULIN
RPE1 TP53-/- BRCA1-/-
B
Input
TOPBP1 IP
BRCA1 FL EV Δ11 FL EV Δ11
115
225
115
80
50
TOPBP1
BRCA1 (FL)
BRCA1 (Δ11)
TUBULIN
RPE1 TP53-/- BRCA1-/-
C
Input
TOPBP1 IP
BRCA1 - EV FL - EV FL
225
80
115
50
BRCA1 (FL)
BRCA1 (Δ11)
TOPBP1
TUBULIN
SUM149PT
D
Input
TOPBP1 IP
BRCA1 FL Δ11 11q FL Δ11 11q
225
115
80
115
50
BRCA1 (FL)
BRCA1 (11q)
BRCA1 (Δ11)
TOPBP1
TUBULIN
mES
E
Input
TY-1 IP
53BP1-/-
53BP1-/-
BRCA1 EV FL Δ11 EV FL Δ11 EV FL Δ11 EV FL Δ11
225
80
115
50
BRCA1 (FL)
BRCA1 (Δ11)
TOPBP1
TUBULIN
RPE1 TP53-/- BRCA1-/-
F
BRCA1-R1699Q Interactors
-Log p
10.0
7.5
5.0
2.5
0.0
BRCA1
HIST2H2AC
BRCA2
BARD1
CtIP
PALB2
TOPBP1
UIMC1
ABRAXAS
BRIP1
0
5
10
Fold change R1699Q/EV (Log2)
G
DAPI
TY
MDC1
Merge
+EV
+WT
+Δ11
+Δ11.4
+SA
TP53-/- BRCA1-/- + BRCA1
H
DAPI
FLAG-TOPBP1
DAPI
FLAG-TOPBP1
WT
Δ1-2
Δ3
Δ4-5
Δ6
Δ7-8
TP53-/- BRCA1-/- + BRCA1WT + TOPBP1
I
Input
TY-1 IP
- FL 1-2 3 4-5 6 7-8 - FL 1-2 3 4-5 6 7-8
TOPBP1 ΔBRCT
225
115
50
BRCA1
TOPBP1
TUBULIN
RPE1 TP53-/- BRCA1-/- + BRCA1-WT

Figure EV3. Validation of the interaction between TOPBP1 and BRCA1 exon 11 in different cell systems (related to Figs. 1, 2 and 3).

(**A**) Cell lysates harvested 1 h post 5 Gy IR from RPE1 hTERT *TP53*$^{-/-}$ *BRCA1*$^{-/-}$ cells virally reconstituted with dox-inducible TY-1 tagged BRCA1-FL, BRCA1-Δ11 or an EV control were subjected to TY-1 IP and analyzed by western blotting. (**B**) Cell lysates harvested 1 h post 5 Gy IR from RPE1 hTERT *TP53*$^{-/-}$ *BRCA1*$^{-/-}$ cells virally reconstituted with dox-inducible TY-1 tagged BRCA1-FL, BRCA1-Δ11 or an EV control were subjected to endogenous TOPBP1 IP and analyzed by western blotting. (**C**) SUM149PT cells virally reconstituted with dox-inducible TY-1 tagged BRCA1-FL or EV were subjected to TOPBP1 IP 1 h post 5 Gy IR and analyzed by western blotting. (**D**) Lysates of BRCA1-deficient mES cell lines reconstituted with human BRCA1-FL, -Δ11 or -11q were subjected to TOPBP1 IP 1 h post 5 Gy IR and analyzed by western blotting. (**E**) Cell lysates harvested 1 h post 5 Gy IR from RPE1 hTERT *TP53*$^{-/-}$ *BRCA1*$^{-/-}$ or *TP53*$^{-/-}$ *BRCA1*$^{-/-}$ *53BP1*$^{-/-}$ cells virally reconstituted with dox-inducible TY-1 tagged BRCA1-FL, BRCA1-Δ11 or an EV control were subjected to TY-1 IP and analyzed by western blotting. (**F**) Volcano plots depicting statistical differences in binding proteins identified by MS proteomic analysis upon TY-1 IP of RPE1 hTERT *TP53*$^{-/-}$ *BRCA1*$^{-/-}$ cells virally reconstituted with dox-inducible BRCA1-R1699Q 1 h post 5 Gy IR compared to EV 1 h post 5 Gy IR ($n = 3$ (biological replicates), fold-changes were determined by Perseus software, two-tiered *t* tests, P value < 0.05 was considered significant). (**G**) Representative microscopy images of BRCA1-TY-1 and MDC1 foci from RPE1 hTERT *TP53*$^{-/-}$ *BRCA1*$^{-/-}$ cell lines virally reconstituted with dox-inducible FL BRCA1, BRCA1-Δ11, BRCA1-Δ11.4, BRCA1-SA or EV control 4 h post 10 Gy (scale bar 10 μm). (**H**) Representative microscopy images of FLAG-stained TOPBP1 nuclear localization from RPE1 hTERT *TP53*$^{-/-}$ *BRCA1*$^{-/-}$ cell lines virally reconstituted with dox-inducible FL BRCA1 and FLAG-tagged FL TOPBP1, TOPBP1-Δ1-2, TOPBP1-Δ3, TOPBP1-Δ4-5, TOPBP1-Δ6 or TOPBP1-Δ7-8 (scale bar 10 μm). (**I**) Cell lysates harvested 1 h post 5 Gy IR of RPE1 hTERT *TP53*$^{-/-}$ *BRCA1*$^{-/-}$ cells virally reconstituted with dox-inducible BRCA1-FL and the indicated FLAG-tagged TOPBP1 deletion mutants were subjected to TY-1 IP and analyzed by western blotting. Please note that the TOPBP1 staining also visualizes endogenous TOPBP1. Source data are available online for this figure

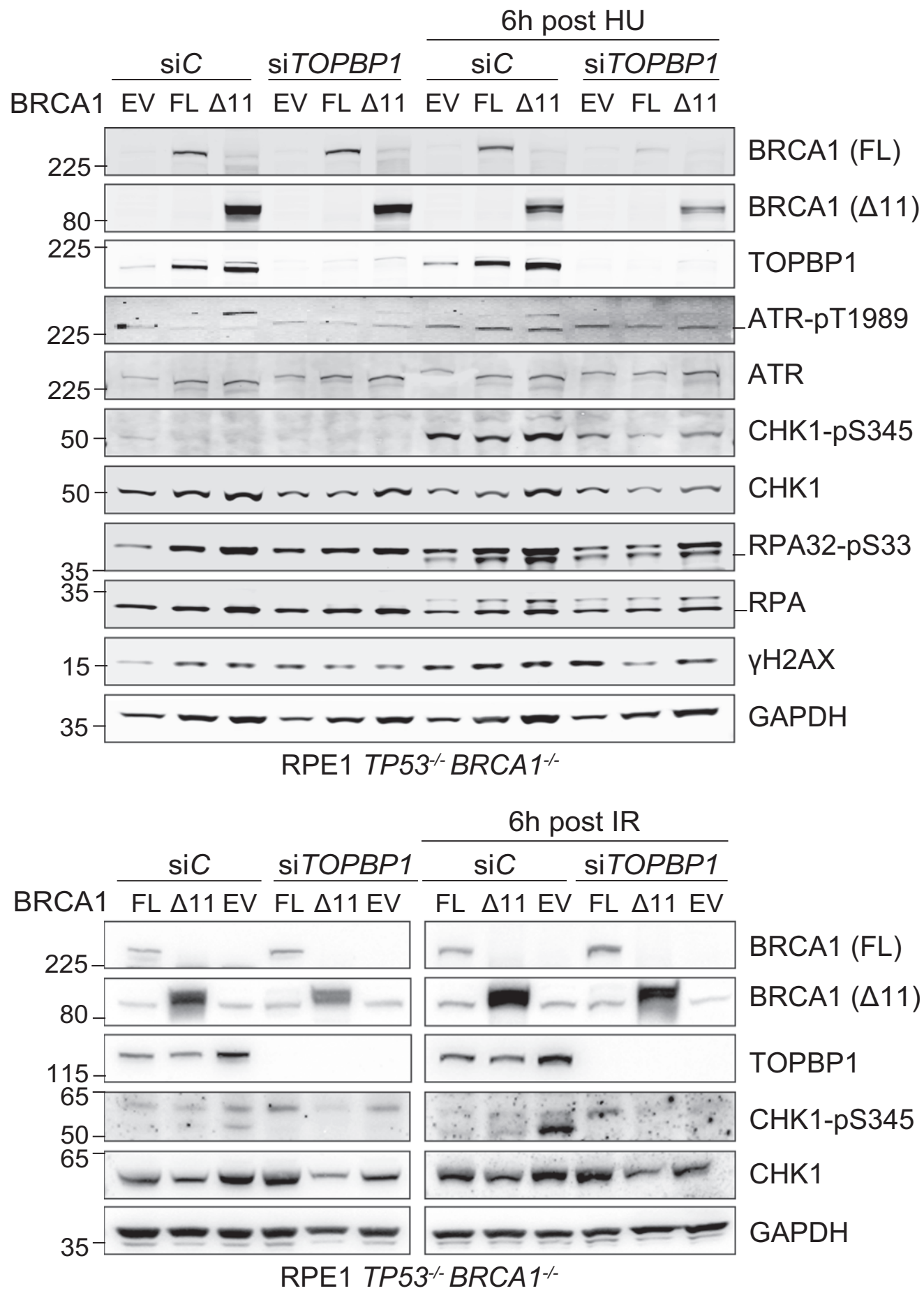

Figure EV4. The interaction between BRCA1 exon 11 and TOPBP1 is not required for checkpoint activation (related to Fig. 4).

Cell lysates from RPE1 hTERT *TP53*$^{-/-}$ *BRCA1*$^{-/-}$ cells virally reconstituted with dox-inducible BRCA1- FL, BRCA1- Δ11 or EV control and transfected with a control (siC) or *TOPBP1*-targetting siRNA (siTOPBP1) harvested 6 h post 20 mM HU treatment (top) or 6 h post 5 Gy IR (bottom) were analyzed by western blotting using the indicated antibodies. Source data are available online for this figure

 © The Author(s)

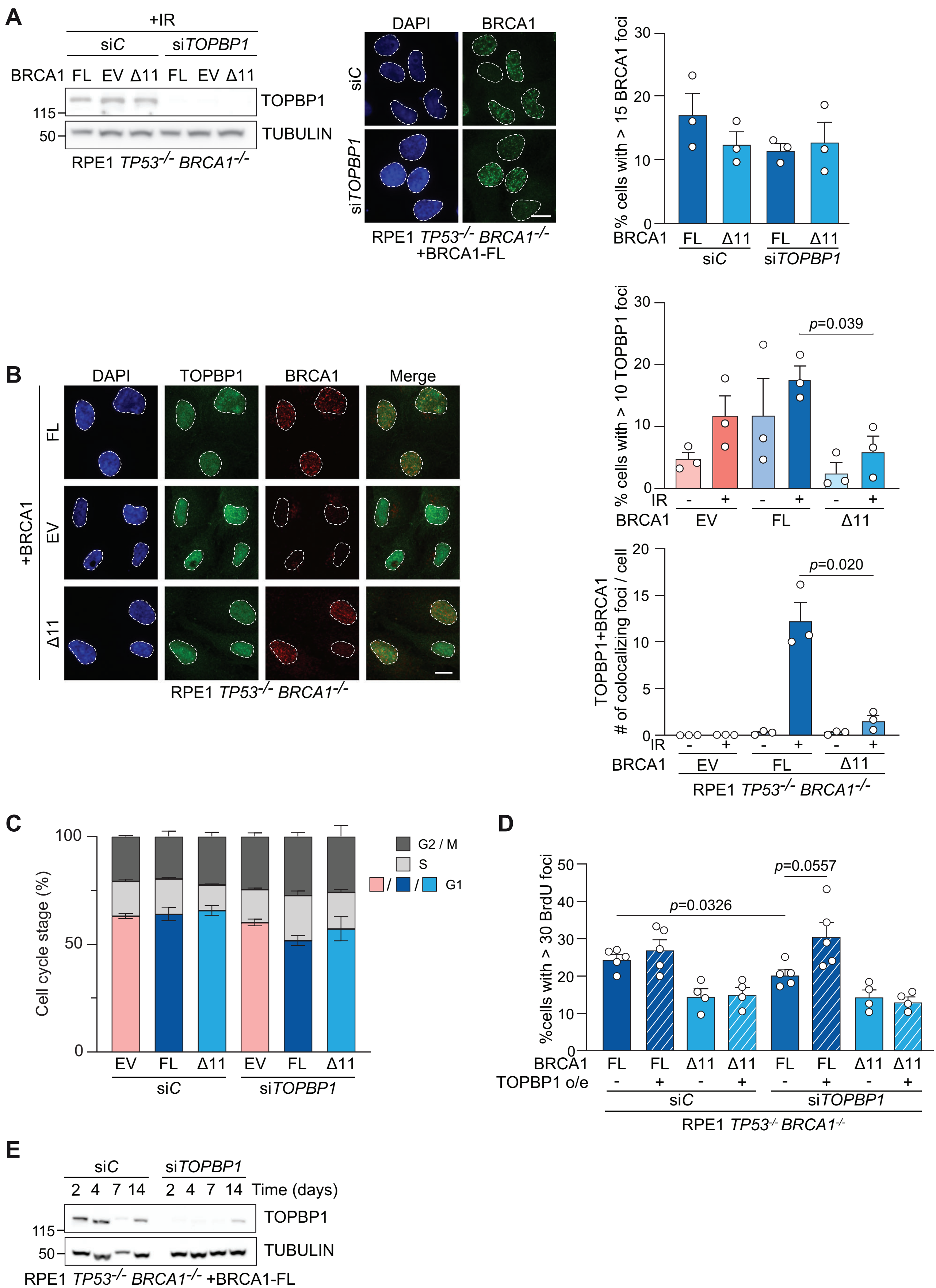
A
+IR
siC
siTOPBP1
BRCA1 FL EV Δ11 FL EV Δ11
TOPBP1
TUBULIN
115
50
RPE1 TP53-/- BRCA1-/-
DAPI
BRCA1
siC
siTOPBP1
RPE1 TP53-/- BRCA1-/- +BRCA1-FL
% cells with > 15 BRCA1 foci
BRCA1 FL Δ11 FL Δ11
siC siTOPBP1
B
DAPI TOPBP1 BRCA1 Merge
+BRCA1
FL
EV
Δ11
RPE1 TP53-/- BRCA1-/-
% cells with > 10 TOPBP1 foci
p=0.039
IR - + - + - +
BRCA1 EV FL Δ11
TOPBP1+BRCA1 # of colocalizing foci / cell
p=0.020
RPE1 TP53-/- BRCA1-/-
C
Cell cycle stage (%)
G2 / M
S
G1
EV FL Δ11 EV FL Δ11
siC siTOPBP1
D
%cells with > 30 BrdU foci
p=0.0557
p=0.0326
BRCA1 FL FL Δ11 Δ11 FL FL Δ11 Δ11
TOPBP1 o/e - + - + - + - +
siC siTOPBP1
RPE1 TP53-/- BRCA1-/-
E
siC siTOPBP1
2 4 7 14 2 4 7 14 Time (days)
TOPBP1
TUBULIN
115
50
RPE1 TP53-/- BRCA1-/- +BRCA1-FL

Figure EV5. The interaction between BRCA1 and TOPBP1 stimulates DNA end-resection (related to Fig. 4).

(**A**) Cell lysates harvested 1 h post 5 Gy IR from RPE1 hTERT *TP53*$^{-/-}$ *BRCA1*$^{-/-}$ cells virally reconstituted with dox-inducible BRCA1-FL, Δ11 or EV control and transfected with a control (siC) or *TOPBP1*-targetting siRNA (siTOPBP1) were analyzed by western blotting to check correct siRNA depletion (left). Representative microscopy images (scale bar 10 µm) (middle) and quantification (right) of BRCA1-TY-1 foci formation 1 h post 5 Gy IR of the same cells ($n = 3$, mean + SEM (biological replicates)). A minimum of 100 cells were counted per replicate. (**B**) Representative microscopy images of TOPBP1 and BRCA1-TY-1 foci from RPE1 hTERT *TP53*$^{-/-}$ *BRCA1*$^{-/-}$ cell lines virally reconstituted with dox-inducible FL BRCA1, an EV control or BRCA1-Δ11 1 h post 5 Gy (scale bar 10 µm). Top right graph shows quantification of TOPBP1 foci ($n = 3$, mean + SEM (biological replicates), paired *t* test, *P* values are shown). A minimum of 100 cells were counted per replicate. Bottom right graph shows TOPBP1 + BRCA1 colocalization ($n = 3$, mean + SEM (biological replicates), paired *t* test, *P* values are shown). A minimum of 25 cells were counted per replicate. (**C**) Cell cycle analysis showing percentages of cells in G1, S and G2/M phase as determined by PI staining and flow cytometry ($n = 3$, mean + SEM (biological replicates)). (**D**) Quantification of BrdU foci from cells described in Fig. 4A ($n = 4$ (cells transduced with BRCA1-Δ11) or 5 (cells transduced with FL BRCA1), mean + SEM, paired *t* test (biological replicates), *P* values are shown). A minimum of 100 cells were counted per replicate. (**E**) Cell lysates from RPE1 hTERT *TP53*$^{-/-}$ *BRCA1*$^{-/-}$ cells virally reconstituted with TY-1 tagged BRCA1-FL transfected with a control (siC) or *TOPBP1*-targetting siRNA (siTOPBP1) were analyzed by western blotting (related to Fig. 4B). Source data are available online for this figure

© The Author(s)
