# Supplementary material for: DNA end-resection is stimulated by an interaction between BRCA1 exon 11 and TOPBP1
Source: EMBO Rep. 2026 Aug 27;27(19):5468–89. doi: 10.1038/s44319-026-00904-3 (PMC13650151; doi:10.1038/s44319-026-00904-3)
Supplement: Supplementary file 2 [file 44319_2026_904_MOESM1_ESM.pdf]

EMBO reports

DNA end resection is stimulated by an interaction between BRCA1 exon 11 and TOPBP1

Marta San Martín Alonso, Rosalie Kampen, Anne Schreuder, Daniel Salas, Arnoud de Ru, Veronica Garzero, Romy Mesman, Román González-Prieto, Peter van Veelen, and Sylvie Noordermeer

Corresponding author(s): Sylvie Noordermeer (s.m.noordermeer@lumc.nl)

Review Timeline:

Submission Date:	2nd Feb 26
Editorial Decision:	8th Apr 26
Revision Received:	9th Jul 26
Editorial Decision:	27th Jul 26
Revision Received:	30th Jul 26
Accepted:	6th Aug 26

Editor: Esther Schnapp

Transaction Report:

(Note: With the exception of the correction of typographical or spelling errors that could be a source of ambiguity, letters and reports are not edited. Depending on transfer agreements, referee reports obtained elsewhere may or may not be included in this compilation. Referee reports are anonymous unless the Referee chooses to sign their reports.)

1st Editorial Decision 8th Apr 2026

Dear Sylvie,

Thank you for the submission of your manuscript with referee reports to EMBO reports. I sent it to 2 new referees, because the previous journal does not share referee identities with us. The comments from both referees are pasted below.

As you will see, the referees acknowledge that the findings are potentially interesting. However, referee 1 also has several suggestions for how the study could be improved and strengthened, and I agree with your revision plan for how to address these points.

I would thus like to invite you to revise your manuscript with the understanding that the referee concerns must be fully addressed and their suggestions taken on board. Please address all referee concerns in a complete point-by-point response.

Acceptance of the manuscript will depend on a positive outcome of a second round of review. It is EMBO reports policy to allow a single round of major revision only and acceptance or rejection of the manuscript will therefore depend on the completeness of your responses included in the next, final version of the manuscript.

We realize that it is difficult to revise to a specific deadline. In the interest of protecting the conceptual advance provided by the work, we recommend a revision within 2 months (9th June 2026). Please discuss the revision progress ahead of this time with us if you require more time to complete the revisions.

You can either publish the study as a short report or as a full article. For short reports, the revised manuscript should not exceed 29,000 characters (including spaces but excluding materials & methods and references) and 5 main plus 5 expanded view figures. The results and discussion sections must further be combined, which will help to shorten the manuscript text by eliminating some redundancy that is inevitable when discussing the same experiments twice. For a normal article there are no length limitations, but it should have more than 5 main figures and the results and discussion sections must be separate. In both cases, the entire materials and methods must be included in the main manuscript file.

IMPORTANT NOTE: we perform an initial quality control of all revised manuscripts before re-review. Your manuscript will FAIL this control and the handling will be DELAYED if the following APPLIES:
1) A data availability section providing access to data deposited in public databases is missing. If you have not deposited any data, please add a sentence to the data availability section that explains that.
2) Your manuscript contains statistics and error bars based on n=2. Please use scatter blots in these cases. No statistics should be calculated if n=2.

When submitting your revised manuscript, please review the submission guidelines (https://link.springer.com/journal/44319/submission-guidelines#cms-Revised-submissions) and carefully review the instructions that follow below. Failure to include requested items will delay the evaluation of your revision.

1) a .docx formatted version of the manuscript text (including legends for main figures, EV figures and tables). Please make sure that the changes are highlighted to be clearly visible.

2) individual production quality figure files as .eps, .tif, .jpg (one file per figure). See hhttps://media.springernature.com/original/springer-cms/rest/v1/content/27825798/data/v1 for more info on how to prepare your figures.

3) We replaced Supplementary Information with Expanded View (EV) Figures and Tables that are collapsible/expandable online. A maximum of 5 EV Figures can be typeset. EV Figures should be cited as 'Figure EV1, Figure EV2" etc... in the text and their respective legends should be included in the main text after the legends of regular figures.

- For the figures that you do NOT wish to display as Expanded View figures, they should be bundled together with their legends in a single PDF file called *Appendix*, which should start with a short Table of Content. Appendix figures should be referred to in the main text as: "Appendix Figure S1, Appendix Figure S2" etc. See 'Expanded View' section of the submission guidelines.

- Additional Tables/Datasets should be labeled and referred to as Table EV1, Dataset EV1, etc. Legends have to be provided in a separate tab in case of .xls files. Alternatively, the legend can be supplied as a separate text file (README) and zipped together with the Table/Dataset file.

4) a .docx formatted letter INCLUDING the reviewers' reports and your detailed point-by-point responses to their comments. As part of the EMBO Press transparent editorial process, the point-by-point response is part of the Review Process File (RPF), which will be published alongside your paper.

5) a complete author checklist, which you can download from our author guidelines. Please insert information in the checklist

that is also reflected in the manuscript. The completed author checklist will also be part of the RPF.

6) Please note that all corresponding authors are required to supply an ORCID ID for their name upon submission of a revised manuscript (), which can be linked to your profile in the manuscript submission system.

7) Before submitting your revision, primary datasets produced in this study need to be deposited in an appropriate public database. Please remember to provide a reviewer password if the datasets are not yet public. The accession numbers and database should be listed in a formal "Data Availability" section placed after Methods. Please note that the Data Availability Section is restricted to new primary data that are part of this study. * Note - All links should resolve to a page where the data can be accessed. *

If your study has not produced novel datasets, please mention this fact in the Data Availability Section.

8) At EMBO Press we ask authors to provide source data for the main manuscript figures. You will receive a separate email with instructions for providing source data with your revised manuscript, including how to upload and organize the files.

9) Our journal also encourages inclusion of *data citations in the reference list* to directly cite datasets that were re-used and obtained from public databases. Data citations in the article text are distinct from normal bibliographical citations and should directly link to the database records from which the data can be accessed. In the main text, data citations are formatted as follows: "Data ref: Smith et al, 2001" or "Data ref: NCBI Sequence Read Archive PRJNA342805, 2017". In the Reference list, data citations must be labeled with "[DATASET]". A data reference must provide the database name, accession number/identifiers and a resolvable link to the landing page from which the data can be accessed at the end of the reference.

10) Regarding data quantification, the following points must be specified in each figure legend:

- the name of the statistical test used to generate error bars and P values,

- the number (n) of independent experiments (please specify technical or biological replicates) underlying each data point,

- the nature of the bars and error bars (s.d., s.e.m.),

- If the data are obtained from n<3, please use scatter blots showing the individual data points.

Discussion of statistical methodology can be reported in the materials and methods section, but figure legends should contain a basic description of n, P and the test applied.

- Please also include scale bars in all microscopy images.

11) The journal requires a statement specifying whether or not authors have competing interests (defined as all potential or actual interests that could be perceived to influence the presentation or interpretation of an article). In case of competing interests, this must be specified in your disclosure statement.

12) All Materials and Methods need to be described in the main text using our 'Structured Methods' format, which is required for all research articles. According to this format, the Methods section includes a separate Reagents and Tools Table file (listing key reagents, experimental models, software and relevant equipment and including their sources and relevant identifiers) and a Methods and Protocols section describing the methods using a step-by-step protocol format. The aim is to facilitate adoption of the methodologies across labs. More information on how to adhere to this format as well as a downloadable template (.docx) for the Reagents and Tools Table can be found in our author guidelines.

We would also welcome the submission of cover suggestions, or motifs to be used by our Graphics Illustrator in designing a cover.

As part of the EMBO publication's Transparent Editorial Process, EMBO reports publishes online a Review Process File (RPF) to accompany accepted manuscripts. This File will be published in conjunction with your paper and will include the referee reports, your point-by-point response and all pertinent correspondence relating to the manuscript.

You are able to opt out of this by letting the editorial office know (emboreports@embo.org). If you do opt out, the Review Process File link will point to the following statement: "No Review Process File is available with this article, as the authors have chosen not to make the review process public in this case."

I look forward to seeing a revised form of your manuscript when it is ready. Please use this link to submit your revision: https://embor.msubmit.net/cgi-bin/main.plex

Best regards,

Esther

Esther Schnapp, PhD
Senior Editor
EMBO reports

Referee #1:

This is an outstanding study that is ready for publication in its current format.

Referee #2:

Overall assessment and summary
This manuscript identifies a DNA damage- and phosphorylation-dependent association between BRCA1 exon 11 and TOPBP1 and proposes that this interaction promotes DNA end resection and homologous recombination, while being dispensable for TOPBP1-driven ATR checkpoint activation. The study addresses an important gap because exon 11 is frequently altered by tumor-associated mutations and splice isoforms, yet its mechanistic contributions remain poorly defined. The work is timely and uses complementary cellular systems (RPE1, SUM149PT, and mES) with multiple resection and HR readouts; however, several key mechanistic points require additional controls and clarification to support the central model.

Major comments

1) Directness and mechanism of the BRCA1-TOPBP1 interaction remain unresolved. The data convincingly show a robust, damage-dependent association, but it is unclear whether exon 11 binds TOPBP1 directly or via bridging factors. Demonstrating direct, phosphorylation-dependent binding in vitro using purified TOPBP1 BRCT1-2 (or relevant fragments) and BRCA1 exon 11 phospho-peptides/protein fragments would substantially strengthen the mechanistic claim and also help interpret the BRCT deletion mapping.

2) Phosphorylation dependence should be strengthened beyond the 4xSQ-to-SA mutant. The 4xSA mutant reduces binding, but it does not identify which SQ site(s) drive the interaction or whether redundancy exists. The manuscript would be strengthened by testing (i) single and/or paired SQ-to-SA mutants, (ii) phosphomimetic substitutions where informative, and/or (iii) ATM/ATR inhibition during IR to determine whether the interaction is kinase-dependent in cells. These additions would better support the conclusion that the interaction is damage- and phospho-regulated.

3) The exon 11 deletion series suggests broad sensitivity of TOPBP1 binding to multiple deletions, raising concerns about indirect effects on folding or accessibility rather than a discrete interface. The rationale for concluding a minimal region at aa 1134-1307 should be clarified, given that several deletions reduce binding. This concern further motivates direct binding assays with defined fragments and phospho-dependence, and/or additional controls demonstrating that the exon 11 deletion constructs retain overall BRCA1 integrity (e.g., representative known interactions and/or nuclear localization and damage recruitment).

4) TOPBP1 BRCT mapping requires stronger controls for mutant expression, stability, localization, and IP efficiency. The conclusion that BRCT1-2 are required relies heavily on the ΔBRCT1-2 construct, but this construct shows markedly reduced recovery in IP. The authors should explicitly show comparable input expression and successful pulldown for each TOPBP1 mutant, and ideally confirm nuclear localization, so the negative interaction result cannot be attributed to poor expression or poor immunoprecipitation.

5) TOPBP1 depletion phenocopies BRCA1-Δ11, but additional cell-cycle and toxicity controls are needed given TOPBP1 essentiality. siTOPBP1 may change S-phase fraction and replication stress, which could influence pRPA and olaparib sensitivity independent of the specific BRCA1 interface. The manuscript would benefit from providing cell-cycle profiles (or EdU incorporation/S-phase fraction) under the depletion conditions used for resection and proliferation assays, to support that the observed phenotypes are not driven by gross cell-cycle shifts.

Minor comments

1) Please report biological replicate numbers consistently for each figure panel and clarify when "n=2/3" reflects biological replicates versus technical repeats.

2) Replace "clonogenical" with "clonogenic."

3) In the IP-MS analysis, justify the statistical thresholds used for volcano plots (including cases where p=0.2 is applied) and provide a rationale for handling multiple testing.

Rebuttal

We thank the reviewers for their positive feedback and are pleased that they recognize the significance of our study and its contribution to the field. Below, we will provide a point-to-point response to the issues raised by reviewer 2 in adherence to the revision plan we discussed with the editor.

Referee 1:
This is an outstanding study that is ready for publication in its current format.

Referee 2:
Overall assessment and summary
This manuscript identifies a DNA damage- and phosphorylation-dependent association between BRCA1 exon 11 and TOPBP1 and proposes that this interaction promotes DNA end resection and homologous recombination, while being dispensable for TOPBP1-driven ATR checkpoint activation. The study addresses an important gap because exon 11 is frequently altered by tumor-associated mutations and splice isoforms, yet its mechanistic contributions remain poorly defined. The work is timely and uses complementary cellular systems (RPE1, SUM149PT, and mES) with multiple resection and HR readouts; however, several key mechanistic points require additional controls and clarification to support the central model.

Major comments
1) Directness and mechanism of the BRCA1-TOPBP1 interaction remain unresolved. The data convincingly show a robust, damage-dependent association, but it is unclear whether exon 11 binds TOPBP1 directly or via bridging factors. Demonstrating direct, phosphorylation-dependent binding in vitro using purified TOPBP1 BRCT1-2 (or relevant fragments) and BRCA1 exon 11 phospho-peptides/protein fragments would substantially strengthen the mechanistic claim and also help interpret the BRCT deletion mapping.

We agree that our data cannot discriminate direct *versus* indirect binding of TOPBP1 and BRCA1 and have referred to this in our manuscript at pages 7 (line 176-180). Initially, we have tried to overexpress exon 11 only in cells to study the interaction in more detail. Unfortunately, the domain by itself seems unstable as we were unable to show expression. We feel it falls outside of the scope of this study to address this issue biochemically.

2) Phosphorylation dependence should be strengthened beyond the 4xSQ-to-SA mutant. The 4xSA mutant reduces binding, but it does not identify which SQ site(s) drive the interaction or whether redundancy exists. The manuscript would be strengthened by testing (i) single and/or paired SQ-to-SA mutants, (ii) phosphomimetic substitutions where informative, and/or (iii) ATM/ATR inhibition during IR to determine whether the interaction is kinase-dependent in cells. These additions would better support the conclusion that the interaction is damage- and phospho-regulated.

Our data suggests redundancy between the four SQ sites as smaller deletion mutants including only one or two SQ sites do not completely inhibit the interaction. Hence, we think testing single or paired mutants will not be informative. We agree that phosphomimetic

substitutions or studies with ATM/ATR inhibitors would have strengthened the data, however, together with the editor we decided this falls beyond the current study.

3) The exon 11 deletion series suggests broad sensitivity of TOPBP1 binding to multiple deletions, raising concerns about indirect effects on folding or accessibility rather than a discrete interface. The rationale for concluding a minimal region at aa 1134-1307 should be clarified, given that several deletions reduce binding. This concern further motivates direct binding assays with defined fragments and phospho-dependence, and/or additional controls demonstrating that the exon 11 deletion constructs retain overall BRCA1 integrity (e.g., representative known interactions and/or nuclear localization and damage recruitment).

The rationale for concluding to a minimal region of aa 1,134-1,307 is that smaller deletions within this domain show redundancy in binding inhibition. We have now clarified this in our revised text (please refer to page 7, lines 193-194).

To confirm that the minimal deletion of aa 1,134-1,307 or mutating the 4 SQ sites did not disrupt overall BRCA1 localisation or folding, we have now included data that these variants are recruited normally to sites of DNA damage (new fig. EV 3g). Moreover, in the figure below, the reviewer can appreciate that the different exon 11 deletion constructs still bind to interaction partners of BRCA1 known to bind to domains outside of exon 11 (BARD1: via RING domain, PALB2: via coiled coil domain, ABRAXAS1: via BRCT domains).

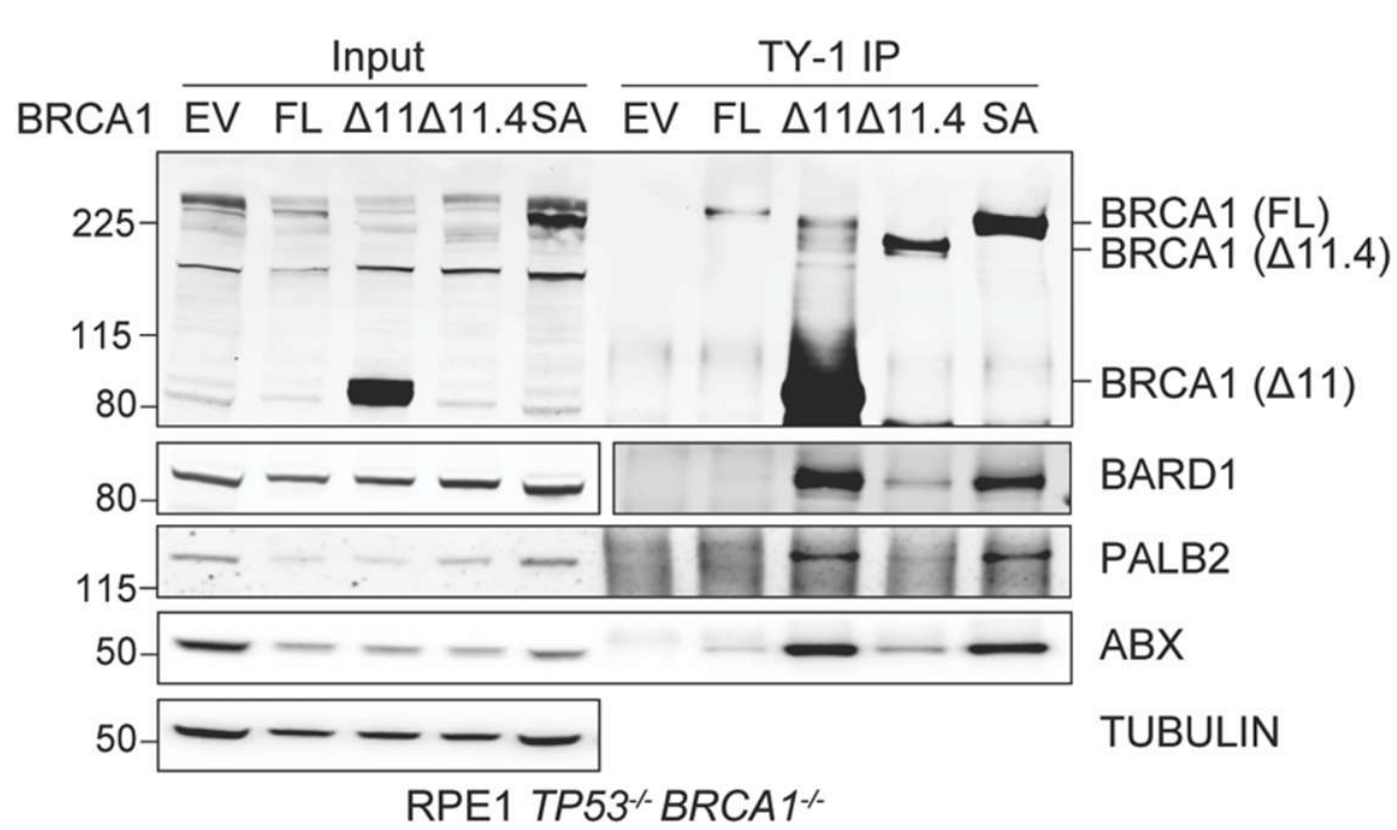

Figure 1 (for reviewer only): IP of different BRCA1-TY1 mutants (BRCA1-full length, BRCA1- Δ11, BRCA1- Δ11.4, BRCA1-SA mutant) showing binding to known interactors outside of the region encoded by exon 11. Unfortunately, expression level differences between the variants have precluded us to equalize IP efficiency (and hence, in this experiment most interactions with full length BRCA1 remain undetected, while we know from other experiments that the interactors also bind full length BRCA1 in our models).

4) TOPBP1 BRCT mapping requires stronger controls for mutant expression, stability, localization, and IP efficiency. The conclusion that BRCT1-2 are required relies heavily on the ΔBRCT1-2 construct, but this construct shows markedly reduced recovery in IP. The authors should explicitly show comparable input expression and successful pulldown for each TOPBP1 mutant, and ideally confirm nuclear localization, so the negative interaction result cannot be attributed to poor expression or poor immunoprecipitation.

By immunofluorescence, we verified nuclear localisation of all TOPBP1 BRCT deletion mutants (new fig. EV 3h), confirming previous research (PMID: 11756551; PMID: 20871591). Moreover, we performed more repeats of the FLAG-IP of the TOPBP1 deletion constructs to improve the IP efficiency (now revised in Fig. 3d). Based on these data, combined with the

data of fig. EV 3i, we have now concluded that BRCT 1-2 deletion result in the strongest defect to BRCA1 binding, but that there is some redundancy with BRCT 4-5 (see revised text on page 8, line 210-219).

5) TOPBP1 depletion phenocopies BRCA1-Δ11, but additional cell-cycle and toxicity controls are needed given TOPBP1 essentiality. siTOPBP1 may change S-phase fraction and replication stress, which could influence pRPA and olaparib sensitivity independent of the specific BRCA1 interface. The manuscript would benefit from providing cell-cycle profiles (or EdU incorporation/S-phase fraction) under the depletion conditions used for resection and proliferation assays, to support that the observed phenotypes are not driven by gross cell-cycle shifts.

We thank the reviewer for this suggestion, as we agree that altered cell cycle progression could cause misinterpretation of our data. By performing cell cycle analyses, we confirmed that the short term TOPBP1 depletion we have used throughout our assays does not decrease S phase entry (as determined by flow cytometry PI staining or immunofluorescence detection of EdU incorporation (new Fig. EV 5c and Figure 2 below for the reviewer's discretion, respectively)). These data are nicely in line with previous literature (*e.g.* PMID: 42011787; PMID: 16880517) and indicate that the reduction in DNA end-resection is not a secondary effect of reduced S-G2 cell cycle progression.

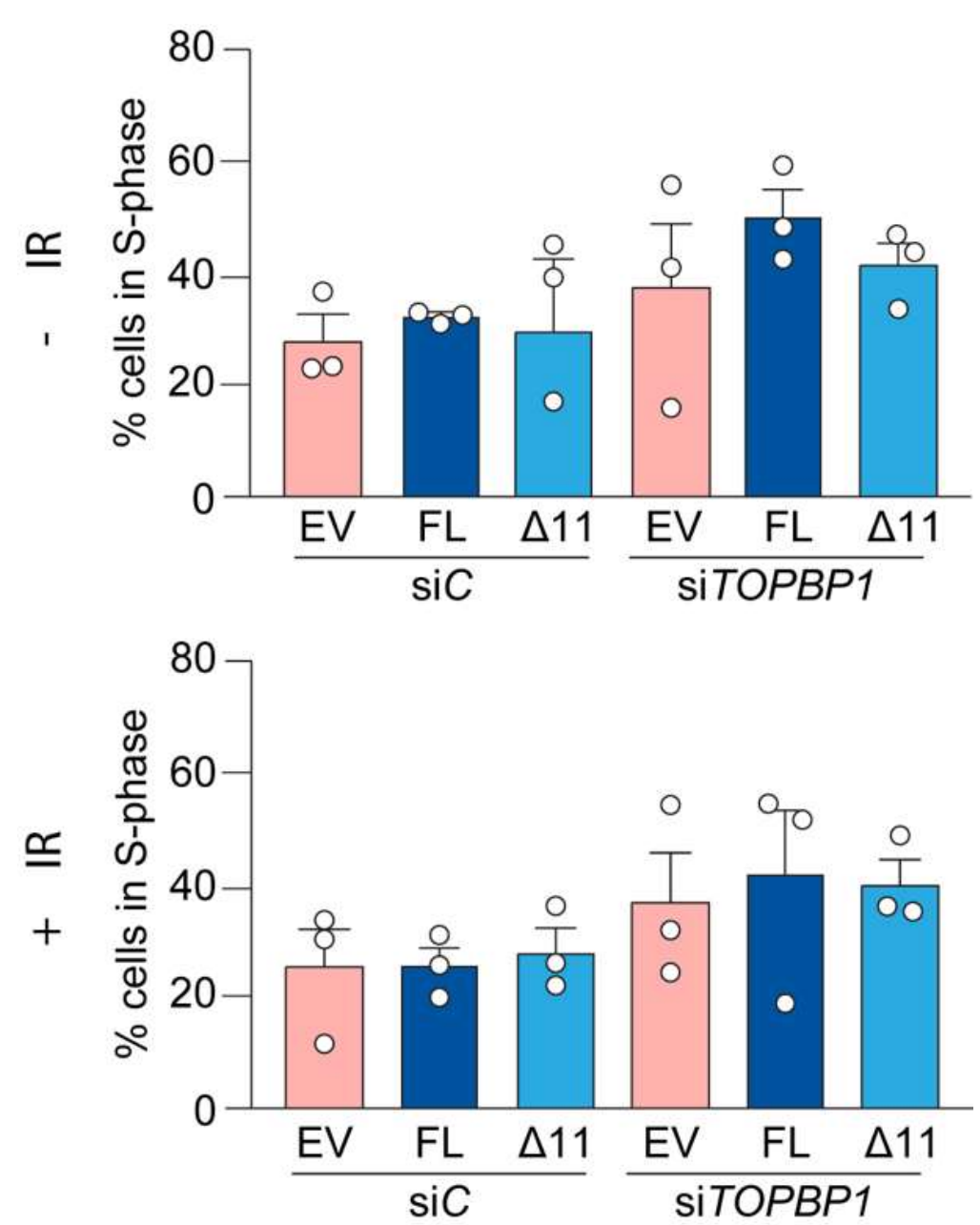

Figure 2 (for reviewer only): S-phase progression is not altered by TOPBP1 depletion. Cells were transfected with siRNAs against TOPBP1 (or control). 48 hr post transfection, cells were pulsed with EdU for 30 min, followed by fixation, Azide-AlexaFluor488 click chemistry and imaging to detect Edu-positive cells

Minor comments

1) Please report biological replicate numbers consistently for each figure panel and clarify when "n=2/3" reflects biological replicates versus technical repeats.

All data reported represents biological replicates. We have now indicated this in the figure legends. Moreover, we have indicated the number of replicates per sample in graphs where replicates vary between conditions (Fig. 1C, Fig. 4A, Fig. EV 5d).

2) Replace "clonogenical" with "clonogenic."

We have ensured using ‘clonogenic’ throughout the text.

3) In the IP-MS analysis, justify the statistical thresholds used for volcano plots (including cases where p=0.2 is applied) and provide a rationale for handling multiple testing.

We thank the reviewer for his/her eye for detail. We agree a p-value of 0.2 is not meaningful and have now changed all statistical tresholds in the volcano plots to 0.05 ($\log_{10}(0.05)=-1.3$). Multiple testing is not common practice in IP-MS as this method is prone for detection of many background proteins. Hence, in our opinion it is preferable to use IP-MS as a first ‘hypothesis generation method’ prioritizing biological replicate consistency and provide dedicated confirmation as follow-up.

Dear Sylvie,

Thank you for the submission of your revised manuscript. We have now received the enclosed report from referee 2 and I am happy to say that s/he supports the publication of your study now. Only a few editorial requests will need to be addressed before we can proceed with the official acceptance of your manuscript:

- Please correct the conflict of interest subheading to "Disclosure and Competing Interests Statement" and place it after the Acknowledgments.

- The author credits need to be removed from the ms file. All credits need to be entered during online ms submission.

- In the author checklist, please answer all questions on statistics and send us a new, completed list.

- All Expanded View (EV) figures need to be uploaded as individual, production quality Figure files. The nomenclature for EV figures is not correct: It should be Figure EV1, etc. in all places

- Figure 2 is not properly converted to PDF, it looks extremely small.

- FIGURE CALLOUTS: "Supplemental Text" or "Supplemental Table" are not correct callouts, please correct.

- DATASET EV LEGENDS: Table EV1 and EV2 are datasets and need to be updated to Dataset EV1 and Dataset EV2 and the names need to be corrected in all places.

- Please upload the Reagents & Tools table as a separate file.

- The Excel file in each Source Data (SD) figure folder needs to be clearly labeled with the panels it contains, e.g. Fig1BCDEFG. Also, we need the URL for the large deposited SD provided in the Data Availability Section (DAS). The specific URL for dataset (PXD079087) is not provided in the DAS.

- "Methods" is the correct title for this section.

- Please indicate the statistical test used for data analysis in the legends of figures 1F,G; 2B; EV-1C; EV-2D; EV-3F.

EMBO press papers are accompanied online by A) a short (1-2 sentences) summary of the findings and their significance, B) 2-3 bullet points highlighting key results and C) a synopsis image that is exactly 550 pixels wide and 300 pixels high. The synopsis image should provide a sketch of the major findings, like a graphical abstract. Please note that text needs to be readable at the final size. Please send us this information along with the final manuscript.

I look forward to seeing a final version of your manuscript as soon as possible. Please use this link to submit your revision: https://embor.msubmit.net/cgi-bin/main.plex

Kind regards,
Esther

Esther Schnapp, PhD
Senior Editor
EMBO reports

Referee #2:

The authors have adequately addressed the major concerns raised in the previous review. I support publication of the manuscript.

All editorial and formatting issues were resolved by the authors.

Dr. Sylvie Noordermeer
Leiden University Medical Center; Oncode Institute
Human Genetics
Netherlands

Dear Dr. Noordermeer,

I am very pleased to accept your manuscript for publication in the next available issue of EMBO reports. Thank you for your contribution to our journal.

Your manuscript will be processed for publication by EMBO Press. It will be copy edited and you will receive page proofs prior to publication. Please note that you will be contacted by Springer Nature Author Services to complete licensing and payment information.

You may qualify for financial assistance for your publication charges - either via a Springer Nature fully open access agreement or an EMBO initiative. Check your eligibility: https://link.springer.com/journal/44319/how-to-publish-with-us

Should you be planning a Press Release on your article, please notify the production team via this form in order to coordinate publication and release dates: https://forms.cloud.microsoft/e/eH4A8JVP6g

If you have any questions, please do not hesitate to contact the Editorial Office. Thank you for your contribution to EMBO Reports.

Best regards,
Esther

Esther Schnapp, PhD
Senior Editor
EMBO reports

>>> Please note that it is EMBO Reports policy for the transcript of the editorial process (containing referee reports and your response letter) to be published as an online supplement to each paper. If you do NOT want this, you will need to inform the Editorial Office via email immediately. More information is available here: https://link.springer.com/partners/embo-press/editorial-policies#Peer%20review

EMBO Press Author Checklist

Corresponding Author Name: Sylvie Noordermeer	
Journal Submitted to: EMBO reports	
Manuscript Number: EMBOR-2026-63738V1	

USEFUL LINKS FOR COMPLETING THIS FORM
The EMBO Journal - Author Guidelines
EMBO Reports - Author Guidelines
Molecular Systems Biology - Author Guidelines
EMBO Molecular Medicine - Author Guidelines

Reporting Checklist for Life Science Articles (updated January
This checklist is adapted from Materials Design Analysis Reporting (MDAR) Checklist for Authors. MDAR establishes a minimum set of requirements in transparent reporting in the life sciences (see Statement of Task: 10.31222/osf.io/9sm4x). Please follow the journal's guidelines in preparing your
Please note that a copy of this checklist will be published alongside your article.

Abridged guidelines for figures
1. Data
The data shown in figures should satisfy the following conditions:
- the data were obtained and processed according to the field's best practice and are presented to reflect the results of the experiments in an accurate and unbiased manner.
- ideally, figure panels should include only measurements that are directly comparable to each other and obtained with the same assay.
- plots include clearly labeled error bars for independent experiments and sample sizes. Unless justified, error bars should not be shown for technical
- if n<5, the individual data points from each experiment should be plotted. Any statistical test employed should be justified.
- Source Data should be included to report the data underlying figures according to the guidelines set out in the authorship guidelines on Data

2. Captions
Each figure caption should contain the following information, for each panel where they are relevant:
- a specification of the experimental system investigated (eg cell line, species name).
- the assay(s) and method(s) used to carry out the reported observations and measurements.
- an explicit mention of the biological and chemical entity(ies) that are being measured.
- an explicit mention of the biological and chemical entity(ies) that are altered/varied/perturbed in a controlled manner.
- the exact sample size (n) for each experimental group/condition, given as a number, not a range;
- a description of the sample collection allowing the reader to understand whether the samples represent technical or biological replicates (including how many animals, litters, cultures, etc.).
- a statement of how many times the experiment shown was independently replicated in the laboratory.
- definitions of statistical methods and measures:
  - common tests, such as t-test (please specify whether paired vs. unpaired), simple χ2 tests, Wilcoxon and Mann-Whitney tests, can be unambiguously identified by name only, but more complex techniques should be described in the methods section;
  - are tests one-sided or two-sided?
  - are there adjustments for multiple comparisons?
  - exact statistical test results, e.g., P values = x but not P values < x;
  - definition of 'center values' as median or average;
  - definition of error bars as s.d. or s.e.m.

Please complete ALL of the questions below.
Select "Not Applicable" only when the requested information is not relevant for your study.

Materials

Newly Created Materials	Information included in the manuscript?	In which section is the information available? (Reagents and Tools Table, Materials and Methods, Figures, Data Availability Section)
New materials and reagents need to be available; do any restrictions apply?	Yes	Reagents and tools table, methods section

Antibodies	Information included in the manuscript?	In which section is the information available? (Reagents and Tools Table, Materials and Methods, Figures, Data Availability Section)
For **antibodies** provide the following information:<br>- Commercial antibodies: RRID (if possible) or supplier name, catalogue number and or/clone number<br>- Non-commercial: RRID or citation	Yes	Reagents and tools table

DNA and RNA sequences	Information included in the manuscript?	In which section is the information available? (Reagents and Tools Table, Materials and Methods, Figures, Data Availability Section)
**Short novel DNA or RNA including primers, probes**: provide the sequences.	Yes	Reagents and tools table

Cell materials	Information included in the manuscript?	In which section is the information available? (Reagents and Tools Table, Materials and Methods, Figures, Data Availability Section)
**Cell lines:** Provide species information, strain. Provide accession number in repository **OR** supplier name, catalog number, clone number, and/**OR** RRID.	Yes	Reagents and tools table
**Primary cultures:** Provide species, strain, sex of origin, genetic modification status.	Not Applicable	
Report if the cell lines were recently **authenticated** (e.g., by STR profiling) and tested for mycoplasma contamination.	Yes	methods section

Experimental animals	Information included in the manuscript?	In which section is the information available? (Reagents and Tools Table, Materials and Methods, Figures, Data Availability Section)
**Laboratory animals or Model organisms:** Provide species, strain, sex, age, genetic modification status. Provide accession number in repository **OR** supplier name, catalog number, clone number, **OR** RRID.	Not Applicable	
**Animal observed in or captured from the field:** Provide species, sex, and age where possible.	Not Applicable	
Please detail **housing and husbandry conditions**.	Not Applicable	

Plants and microbes	Information included in the manuscript?	In which section is the information available? (Reagents and Tools Table, Materials and Methods, Figures, Data Availability Section)
**Plants:** provide species and strain, ecotype and cultivar where relevant, unique accession number if available, and source (including location for collected wild specimens).	Not Applicable	
**Microbes:** provide species and strain, unique accession number if available, and source.	Not Applicable	

Human research participants	Information included in the manuscript?	In which section is the information available? (Reagents and Tools Table, Materials and Methods, Figures, Data Availability Section)
If collected and within the bounds of privacy constraints report on age, sex and gender or ethnicity for all study participants.	Not Applicable	

Core facilities	Information included in the manuscript?	In which section is the information available? (Reagents and Tools Table, Materials and Methods, Figures, Data Availability Section)
If your work benefited from core facilities, was their service mentioned in the acknowledgments section?	Not Applicable	

Design

Study protocol	Information included in the manuscript?	In which section is the information available? (Reagents and Tools Table, Materials and Methods, Figures, Data Availability Section)
If study protocol has been **pre-registered, provide DOI in the manuscript**. For clinical trials, provide the trial registration number **OR** cite DOI.	Not Applicable	
Report the **clinical trial registration number** (at ClinicalTrials.gov or equivalent), where applicable.	Not Applicable	

Laboratory protocol	Information included in the manuscript?	In which section is the information available? (Reagents and Tools Table, Materials and Methods, Figures, Data Availability Section)
Provide DOI OR other citation details if **external detailed step-by-step protocols** are available.	Not Applicable	

Experimental study design and statistics	Information included in the manuscript?	In which section is the information available? (Reagents and Tools Table, Materials and Methods, Figures, Data Availability Section)
Include a statement about **sample size** estimate even if no statistical methods were used.	Yes	methods section
Were any steps taken to minimize the effects of subjective bias when allocating animals/samples to treatment (e.g. **randomization procedure**)? If yes, have they been described?	Not Applicable	
Include a statement about **blinding** even if no blinding was done.	Yes	methods section
Describe **inclusion/exclusion criteria** if samples or animals were excluded from the analysis. Were the criteria pre-established?<br><br>If sample or data points were omitted from analysis, report if this was due to attrition or intentional exclusion and provide justification.	Not Applicable	
For every figure, are **statistical tests** justified as appropriate? Do the data meet the assumptions of the tests (e.g., normal distribution)? Describe any methods used to assess it. Is there an estimate of variation within each group of data? **I**s the variance similar between the groups that are being statistically compared?	Yes	figure legends

Sample definition and in-laboratory replication	Information included in the manuscript?	In which section is the information available? (Reagents and Tools Table, Materials and Methods, Figures, Data Availability Section)
In the figure legends: state number of times the experiment was **replicated** in laboratory.	Yes	figure legends
In the figure legends: define whether data describe **technical or biological replicates**.	Yes	figure legends

Ethics

Ethics	Information included in the manuscript?	In which section is the information available? (Reagents and Tools Table, Materials and Methods, Figures, Data Availability Section)
Studies involving **human participants**: State details of **authority granting ethics approval** (IRB or equivalent committee(s), provide reference number for approval.	Not Applicable	
Studies involving **human participants**: Include a statement confirming that **informed consent** was obtained from all subjects and that the experiments conformed to the principles set out in the WMA Declaration of Helsinki and the Department of Health and Human Services Belmont Report.	Not Applicable	
Studies involving **human participants:** For publication of **patient photos**, include a statement confirming that consent to publish was obtained.	Not Applicable	
Studies involving experimental **animals**: State details of **authority granting ethics approval** (IRB or equivalent committee(s), provide reference number for approval. Include a statement of compliance with ethical regulations.	Not Applicable	
Studies involving **specimen and field samples:** State if relevant **permits** obtained, provide details of authority approving study; if none were required, explain why.	Not Applicable	

Dual Use Research of Concern (DURC)	Information included in the manuscript?	In which section is the information available? (Reagents and Tools Table, Materials and Methods, Figures, Data Availability Section)
Could your study fall under dual use research restrictions? Please check biosecurity documents and list of **select agents and toxins** (CDC): https://www.selectagents.gov/sat/list.htm	Not Applicable	
If you used a select agent, is the security level of the lab appropriate and reported in the manuscript?	Not Applicable	
If a study is subject to dual use research of concern regulations, is the name of the **authority granting approval and reference number** for the regulatory approval provided in the manuscript?	Not Applicable	

Reporting

The MDAR framework recommends adoption of discipline-specific guidelines, established and endorsed through community initiatives. Journals have their own policy about requiring specific guidelines and recommendations to complement MDAR.

Adherence to community standards	Information included in the manuscript?	In which section is the information available? (Reagents and Tools Table, Materials and Methods, Figures, Data Availability Section)
State if relevant guidelines or checklists (e.g., **ICMJE, MIBBI, ARRIVE, PRISMA**) have been followed or provided.	Not Applicable	
For **tumor marker prognostic studies,** we recommend that you follow the **REMARK** reporting guidelines (see link list at top right). See author guidelines, under 'Reporting Guidelines'. Please confirm you have followed these guidelines.	Not Applicable	
For **phase II and III randomized controlled trials**, please refer to the **CONSORT** flow diagram (see link list at top right) and submit the CONSORT checklist (see link list at top right) with your submission. See author guidelines, under 'Reporting Guidelines'. Please confirm you have submitted this list.	Not Applicable	

Data Availability

Data availability	Information included in the manuscript?	In which section is the information available? (Reagents and Tools Table, Materials and Methods, Figures, Data Availability Section)
Have **primary datasets** been deposited according to the journal's guidelines (see 'Data Deposition' section) and the respective accession numbers provided in the Data Availability Section?	Yes	data availability section
Were **human clinical and genomic datasets** deposited in a public access-controlled repository in accordance to ethical obligations to the patients and to the applicable consent agreement?	Not Applicable	
Are **computational models** that are central and integral to a study available without restrictions in a machine-readable form? Were the relevant accession numbers or links provided?	Not Applicable	
If publicly available data were reused, provide the respective **data citations in the reference list**.	Not Applicable	
