# Supplementary material for: DNA end-resection is stimulated by an interaction between BRCA1 exon 11 and TOPBP1
Source: EMBO Rep. 2026 Aug 27;27(19):5468–89. doi: 10.1038/s44319-026-00904-3 (PMC13650151; doi:10.1038/s44319-026-00904-3)

Fig. EV5 San Martin Alonso *et al.*

a

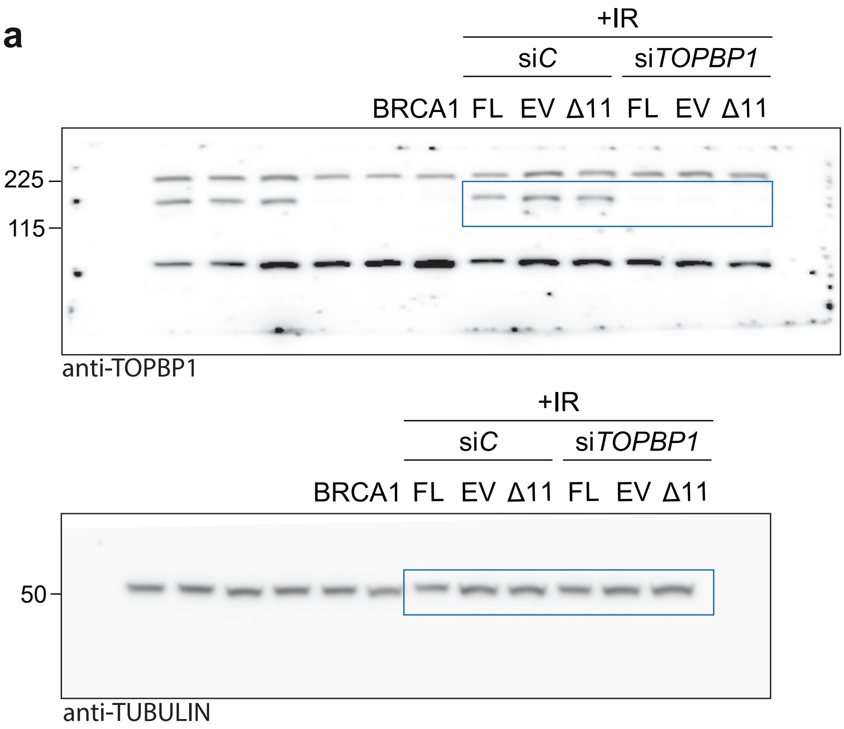

e

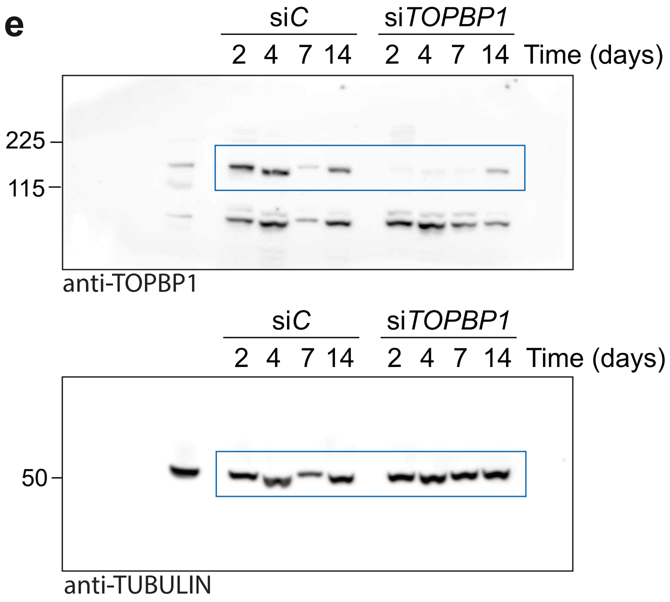

Supplement: Supplementary file 5 [file 44319_2026_904_MOESM12_ESM.zip › Figure EV5/EV5AE_WB.pdf]
