# Supplementary material for: DNA end-resection is stimulated by an interaction between BRCA1 exon 11 and TOPBP1
Source: EMBO Rep. 2026 Aug 27;27(19):5468–89. doi: 10.1038/s44319-026-00904-3 (PMC13650151; doi:10.1038/s44319-026-00904-3)

Fig. EV2 San Martin Alonso *et al.*

b

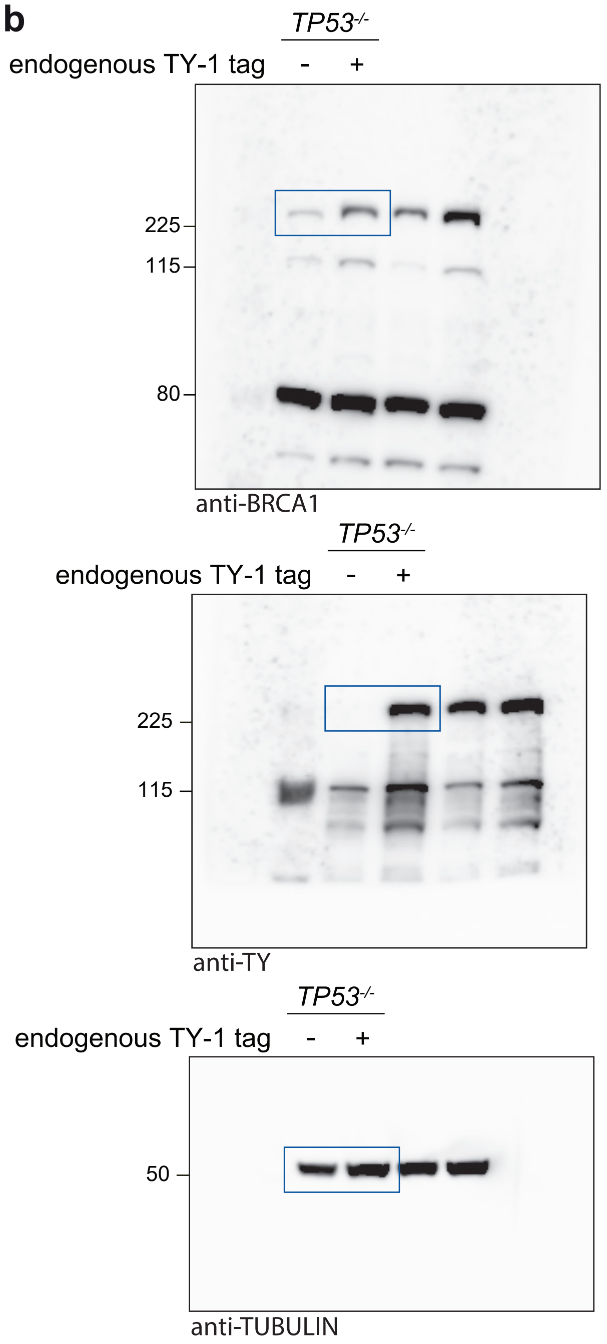

Supplement: Supplementary file 11 [file 44319_2026_904_MOESM9_ESM.zip › Figure EV2/EV2B_WB.pdf]
